# High Milk Somatic Cell Counts and Increased *Teladorsagia* Burdens Overshadow Non-Infection-Related Factors as Predictors of Fat and Protein Content of Bulk-Tank Raw Milk in Sheep and Goat Farms

**DOI:** 10.3390/foods11030443

**Published:** 2022-02-02

**Authors:** Daphne T. Lianou, Charalambia K. Michael, Dimitris A. Gougoulis, Peter J. Cripps, Natalia G. C. Vasileiou, Nikolaos Solomakos, Efthymia Petinaki, Angeliki I. Katsafadou, Elisavet Angelidou, Konstantinos V. Arsenopoulos, Elias Papadopoulos, Marzia Albenzio, Vasia S. Mavrogianni, Mariangela Caroprese, George C. Fthenakis

**Affiliations:** 1 Veterinary Faculty, University of Thessaly, 43100 Karditsa, Greece; dlianou@vet.uth.gr (D.T.L.); cmichail@vet.uth.gr (C.K.M.); dgoug@vet.uth.gr (D.A.G.); peterjohncripps@gmail.com (P.J.C.); nsolom@vet.uth.gr (N.S.); eaggel@vet.uth.gr (E.A.); vmavrog@vet.uth.gr (V.S.M.); 2 Faculty of Animal Science, University of Thessaly, 41110 Larissa, Greece; vasileiounat@gmail.com; 3 University Hospital of Larissa, 41110 Larissa, Greece; petinaki@uth.gr; 4 Faculty of Public and One Health, University of Thessaly, 43100 Karditsa, Greece; agkatsaf@vet.uth.gr; 5 Laboratory of Parasitology and Parasitic Diseases, School of Veterinary Medicine, Faculty of Health Sciences, Aristotle University of Thessaloniki, 54124 Thessaloniki, Greece; arsenopo@vet.auth.gr (K.V.A.); eliaspap@vet.auth.gr (E.P.); 6 Department of Agriculture, Food, Natural Resources and Engineering (DAFNE), University of Foggia, 71122 Foggia, Italy; marzia.albenzio@unifg.it (M.A.); mariangela.caroprese@unifg.it (M.C.)

**Keywords:** bulk tank, fat content, goat, mastitis, milk composition, protein content, raw milk, sheep, somatic cell counts, *Teladorsagia*

## Abstract

This paper presents the results of an extensive countrywide investigation performed in 325 dairy sheep flocks and 119 goat herds throughout Greece. The objectives of the study were (a) to investigate fat and protein content in the bulk-tank raw milk of small ruminant farms in Greece and (b) to identify factors potentially influencing that content and factors that can contribute to increased content. The mean fat/protein contents in bulk-tank raw milk of sheep and goats were 6.16 ± 0.05%/4.43 ± 0.01% and 4.77 ± 0.44%/3.23 ± 0.30%, respectively. Significant differences were seen in protein content between farms in the various parts of the country. For sheep, multivariable analyses revealed breed and age of lamb removal from dams as significant for fat content, and somatic cell counts, management system in the farm, administration of anthelmintic treatment during the last stage of pregnancy, and farmer education as significant for protein content. For goats, significant factors were month into lactation period, age of kid removal from dams, and breed for fat content, and somatic cell counts, month into lactation, grazing duration, and % *Teladorsagia* larvae in faecal samples for protein content. For concurrently high fat and protein content, in multivariable analyses, the following emerged as significant factors: somatic cell counts in milk, numbers of parasite eggs in faeces, and veterinary collaboration (sheep), and month into lactation and somatic cell counts in milk (goats). The results indicate that high somatic cell counts in milk (reflecting the presence of mastitis) and gastrointestinal parasitic infections (mainly *Teladorsagia* infection) appear to exert a more significant influence on fat and protein content of milk, in comparison to non-infection-related factors.

## 1. Introduction

Greece has a high number of sheep and goats, around 8,400,000 sheep and 3,600,000 goats [1], which account for approximately 6.5% and 22.0%, respectively, of total numbers of small ruminants in Europe [2]. The respective milk production from these animals amounts to 645,000 and 350,000 tonnes annually [2,3], 90% of which is used for cheese production.

Milk from sheep and goats is of particular significance for the Greek agricultural sector because of its increased use in dairy products. Annual cheese production from sheep and goat milk varies from 180,000 to 200,000 tonnes. In total, 22 cheese types produced from sheep and/or goat milk in the country have been characterised as protected designation of origin [4]. This puts additional requirements on the quality of raw milk to be used for production of these cheese types. For example, standards for production of “feta” cheese indicate that the minimum fat content of raw milk should be 6% [5]. In view of those requirements, dairy industries in the country have included the fat and total protein composition of the raw milk in the calculation of the raw milk purchase price, given that these two parameters in raw milk can affect cheese yield, as well as the quality of the final product [6]. Moreover, the fat and protein content of raw milk contribute, to a large extent, to the nutrient value of cheese [7,8,9] and, hence, there is a scope to assess the possibilities for improvement. The above also indicate the international significance of milk production from small ruminants in Greece, since a large proportion (25% [10]) of the cheese manufactured is exported to global markets. Nevertheless, systematic countrywide investigations into the composition of the bulk-tank raw milk of sheep and goats in the country have never been reported.

Sheep and goat milk and dairy products have been associated with marked health benefits for their consumers. They can be sources of bioactive molecules (e.g., fatty acids, immunoglobulins, vitamins, minerals) with health-promoting effects to consumers. They also contain various biopeptides with a variety of functions [11,12,13,14,15].

Various papers concerning the composition of milk of small ruminants have been published internationally. A search on the platform Web of Science using the terms [*milk*] AND [*composition* OR *content*] AND [*sheep* OR goat*] revealed a total of 5305 relevant papers (5134 research articles and 171 reviews) published from 1970 to the end of 2021. The authors of these papers were based in 119 countries, among which Spain (*n* = 717 papers), Italy (*n* = 705 papers), United States of America (*n* = 459 papers), France (*n* = 379 papers), and Brazil (*n* = 352 papers) were more frequent. A list of the 32 more frequently cited (>10.0 citations per year after publication) relevant papers among the above is given in Table A1.

Studies on factors that could affect the composition of raw milk have traditionally focused on husbandry-related variables prevailing in the farm and, in this context, a lot of research has been published regarding milk composition of the various breeds of animals. For example, Sakul and Boylan [16] have evaluated the milk composition of 10 sheep-breeds in the USA, whilst Bencini and Pulina [17] have reviewed relevant studies in 29 European sheep breeds. Moreover, for goats, Amills et al. [18] have reviewed research regarding milk composition of 10 European breeds. These differences in milk composition of the various breeds reflect the varying genetic background of the animals [19]. However, in commercial farm settings, there is little that can be done to alter the group’s genetics after breed selection has been finalised and the animals have been brought into a farm.

Given the above, nutritional manipulations can be a further important tool to regulate the composition of milk. Fat is more sensitive to nutrition-related modifications than protein [20]. In this respect, supplementation of dairy animals with protected fats may enhance the concentration of unsaturated fatty acids in the milk. Moreover, regulation of forage and concentrates provided to animals and of the amount and source of dietary protein and fat are considered important determinants of protein content in milk [20]. In all cases of attempted nutritional manipulation, it is important to identify both differences in responses that affect fat and protein content (i.e., % in milk) and those affecting their yield (i.e., total fat and protein output per day) [21].

This paper presents the results of an extensive countrywide investigation performed in 325 dairy sheep flocks and 119 goat herds throughout Greece. The objectives of the study were (a) to investigate fat and protein content in the bulk-tank raw milk of small ruminant farms in Greece and (b) to identify factors potentially influencing that content and factors that can contribute to increased content.

## 2. Materials and Methods

### 2.1. Sheep and Goat Farms

**Figure 1:**
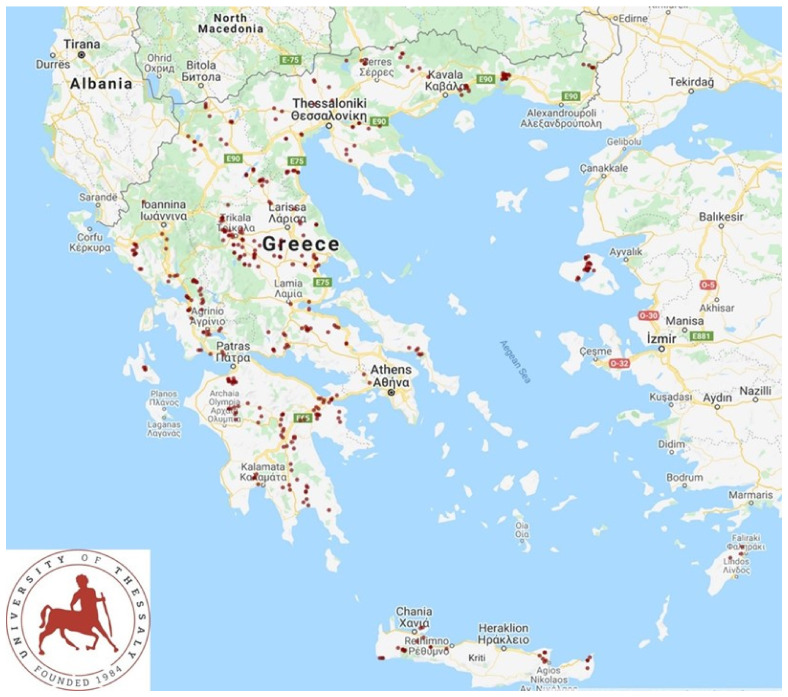
Location of 444 small ruminant farms around Greece, visited during a countrywide investigation in Greece.

From April 2019 to July 2020, a cross-sectional study was performed, in which 325 dairy sheep flocks and 119 dairy goat herds were included. The farms were located in all the 13 administrative regions of Greece (Figure 1). Full details of the procedures for the selection of and visits to farms have been provided by Lianou et al. [22,23]

### 2.2. Samplings

During the visit to each farm, four 20 mL milk samples were collected by withdrawing milk directly from the bulk-tank milk and always employing aseptic sampling techniques. Of these milk samples, two were used for composition measurement and somatic cell counting and two were used for bacteriological examinations.

Faecal samples were collected directly from the rectum of female animals (ewes and does) in the farm. In each farm, 20, 30, 40, or 50 animals in the milking period (for farms with 165, 166–330, 331–500, or >500 females, respectively) were selected for sampling. For the selection of animals to sample, the ewes or does were walked into the milking area and the necessary number of animals was selected as they walked therein by using an electronic random number generator (www.randomresult.com).

Samples were stored at 0.0–4.0 °C (milk) or at 8.0–10.0 °C (faeces) by using portable refrigerators. Measurements for milk composition and somatic cell counting were performed on each of the samples within 4 h after sample collection. Transportation of the samples to the laboratory was made by the investigators and by car; samples collected from farms in the islands were also transported as accompanying luggage by airplane (Crete, Lesvos, and Rhodes) or by boat (Cephalonia).

### 2.3. Laboratory Examinations

Of the four raw milk samples collected from the bulk tank of each farm, two were used for measurement of milk composition and somatic cell counting. The other two samples were used for the bacteriological examinations. From each of the four samples, two subsamples were created and processed; therefore, each separate test was performed four times (each one in different subsamples).

Initially, measurement of milk composition (Lactoscan Farm Eco; Milkotronic Ltd., Nova Zagora, Bulgaria) and somatic cell counting (Lactoscan SCC; Milkotronic Ltd., Nova Zagora, Bulgaria) were performed on each of the four relevant subsamples. Bacteriological examinations were carried out within 24 h after the collection of samples; total bacterial counts (TBC) were obtained by following the standardised procedures of the American Public Health Association [24], and culturing for isolation and identification of staphylococcal species was performed as described in detail previously [23].

For parasitological examinations, 5 g of each of the faecal samples collected from a farm were taken and mixed to form the pooled faecal sample of the flock/herd, which was then processed in a homogenising blender. The McMaster technique and coproculturing were applied to material from this pooled sample [25].

### 2.4. Data Managament and Analysis

#### 2.4.1. Data Management

The results of the two subsamples that were produced from each of the two milk samples collected from the bulk tank were averaged, and then the two means were again averaged for the final result regarding each bulk-tank milk. This was applied during the processing of samples for chemical composition, somatic cell counting, and total bacterial counting.

For the evaluation of regional differences within the country, four area clusters were created: Central, Islands, North, and South.

For the analyses, the somatic cell counts (SCC) and the total bacterial counts were transformed as described previously [23]. Further, total bacterial counts were transformed to log_10_ and the transformed data were used in the analyses. For the evaluation of epg counts and the proportion of *Teladorsagia* larvae in sheep faecal samples, two (≤300 epg and ≥350 epg) and three (0%, 1–63%, and ≥64) categories, respectively, were created. These categories were based on the following criteria: (a) the threshold for performing anthelmintic treatments in Greece had been previously found to be 320 epg [26] and (b) the average proportion of *Teladorsagia* in sheep faecal samples was 63.5%. The same approach was used for goat faecal samples, but, for the proportion of *Teladorsagia* larvae in faecal samples, the three categories used were 0%, 1–64%, and ≥65%, given that the average proportion of *Teladorsagia* in goat faecal samples was 64.5%.

#### 2.4.2. Statistical Analysis

Data were entered into Microsoft Excel and analysed using SPSS v. 21 (IBM Analytics, Armonk, NY, USA). Basic descriptive analysis was performed. Exact binomial confidence intervals (CI) were obtained.

Differences in fat and protein content of bulk-tank raw milk between the four parts of the country were assessed by using analysis of variance.

The potential association of fat and protein composition of bulk-tank raw milk with somatic cell counts or total bacterial counts was assessed by using analysis of correlation. Milk fat and protein content of farms in which Staphylococci were recovered from that milk were compared to that of farms from which no Staphylococci were recovered by using analysis of variance. Analysis of variance was employed to assess differences in fat and protein composition between the categories of epg counts and proportion of *Teladorsagia* larvae in faecal samples.

In total, 37 husbandry-related variables (referring to infrastructure, animals, production characteristics, and health management in the farms) were evaluated for potential association with fat and protein content in the bulk-tank milk of these farms (Table S1); the details were taken directly from the answers of the interview performed at the start of the visit or calculated based on these answers. For each variable, categories were created according to the answers of the farmers. Fat and protein content in the bulk-tank milk from the farms were compared between the categories of each variable by using one-way analysis of variance. The same procedure was then repeated with 6 human-resources-related variables for potential association with fat and protein composition in the bulk-tank milk of these farms (Table S2).

Subsequently, multivariable models were created using standard (“least-squares”) regression, initially offering to the model all variables that achieved a significance of *p* < 0.2 in the preceding univariable analyses and also that were statistically independent of each other. Separate models were constructed for fat and protein and for sheep and goat farms (Table S3). Variables were removed from the initial model by backwards elimination. The *p* value of removal of a variable was assessed and, for those with a *p* value of >0.2, the variable with the largest probability was removed. This process was repeated until no variable could be removed with a *p* value of >0.2. The variables required for the final multivariable tests for each model are shown in Table S3.

Finally, the outcome of “fat and protein content in bulk-tank raw milk concurrently above the average contents found for all flocks/herds” was considered. For this analysis, only variables that had achieved a significance of *p* < 0.2 in the previous analyses (for association with fat or protein composition) were taken into account, i.e., in total, 31 variables for sheep farms and 19 variables for goat farms. Initially, the importance of predictors was assessed in univariable analyses as appropriate. Separate models were constructed for sheep and goat farms. Based on the results of the univariable analyses, multivariable models were constructed and performed as described above, with the *p* value of removal of a variable being assessed by the likelihood ratio test. The variables required for the final multivariable tests for each model are presented in Table S3.

In all analyses, statistical significance was defined at *p* ≤ 0.05.

## 3. Results

### 3.1. Fat and Protein Content of Bulk-Tank Raw Milk

**Figure 2:**
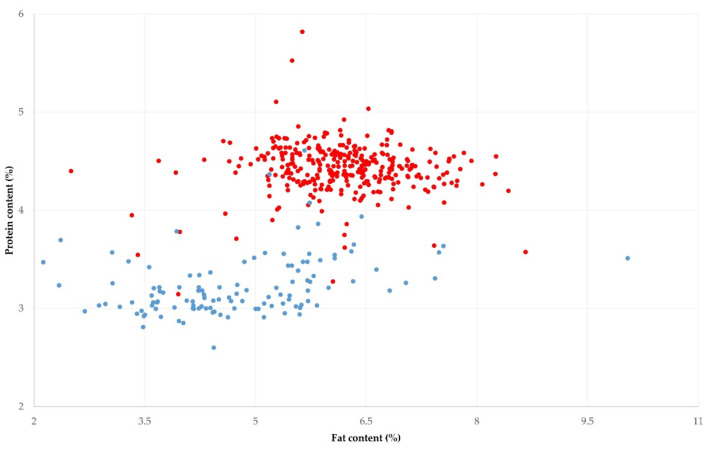
Distribution of fat and protein content in bulk-tank raw milk in 325 sheep (red) and 119 goat (blue) farms, sampled during a countrywide investigation in Greece.

The mean fat and protein content in the bulk-tank raw milk of the 325 sheep flocks visited throughout Greece was 6.16% ± 0.05% and 4.43% ± 0.01% (Table 1, Figure 2). In 79 flocks (24.3%, 95% CI: 20.0–29.3%), fat and protein content concurrently above these means was recorded in the bulk-tank milk.

The mean of the fat and protein in the bulk-tank raw milk of the 119 goat herds visited throughout Greece and sampled was 4.77% ± 0.44% and 3.23% ± 0.30% (Table 1, Figure 2). In 32 herds (26.9%, 95% CI: 17.9–35.5%), fat and protein concurrently above these means were recorded in the bulk-tank milk.

There was a greater variability in the fat than the protein content in the bulk-tank raw milk for both and goat milk (Table 1). There were also significant differences between the four parts of the country in the protein content of the bulk-tank milk for sheep and goats (Table 2).

### 3.2. Association of Milk Somatic Cell Counts and Bacteria in Bulk-Tank Raw Milk with Fat and Protein Content

**Figure 3:**
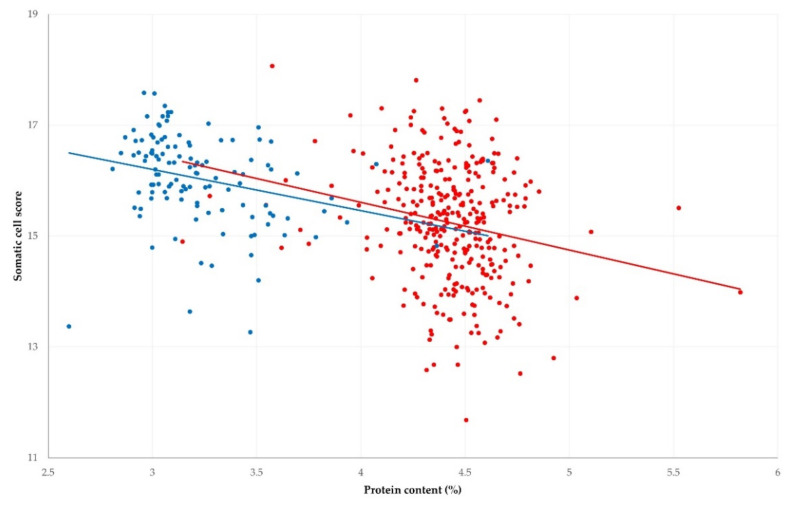
Correlation between somatic cell scores and protein content in bulk-tank raw milk in 325 sheep (red) and 119 goat (blue) farms sampled during a countrywide investigation in Greece (solid lines show trendline slopes).

An inverse correlation was found between increased somatic cell counts and protein content in bulk-tank raw milk from sheep and goats (*r* = −0.211 and −0.280, respectively); this was statistically significant (*p* ≤ 0.001 for both comparisons) (Figure 3). There was no significant difference between the slopes of the associations in sheep and goat milk (*t*-value = 0.388; *p* = 0.70). An inverse correlation was also found between increased somatic cell counts and fat content in milk from sheep (*r* = −0.104; *p* = 0.036), but not from goats (*r* = −0.091; *p* = 0.16).

With regard to bacteria in the milk, neither total bacterial counts nor the identification of staphylococci were significantly associated with fat and protein in bulk-tank raw milk from sheep and goats (*p* ≥ 0.09 for all comparisons).

### 3.3. Association of Parasite Presence in Faecal Samples with Fat and Protein Content in Bulk-Tank Raw Milk

A significantly lower protein content was found in the bulk-tank milk from farms, in which the proportion of *Teladorsagia* larvae in faecal samples was ≥63% (sheep) (*p* = 0.002) or ≥64% (goats) (*p* < 0.0001) (Table 3). No such association was evident for fat content (*p* > 0.23 for all comparisons). There was no significant association between fat and protein in bulk-tank milk and epg counts for both sheep and goats (*p* > 0.065 for all comparisons).

### 3.4. Association of Husbandry- and Human-Resources-Related Variables with Fat and Protein Content in Bulk-Tank Raw Milk

In the univariable analyses, 13 husbandry-related variables were found with a potentially significant effect on fat and protein content in bulk-tank raw milk. Of these, eight were found to have a significant association with % fat and nine with % protein (Table 4, Tables S4–S7). Further, three human-resources-related variables were found with a potentially significant effect on the composition of bulk-tank raw milk (Table 5, Tables S4–S7). The milking system pressure and the yearly length of provision of concentrate feed to adult animals were found to have a significant association with % fat in sheep and goat milk. The month into the lactation period at sampling was significantly associated with % protein in sheep and goat milk.

### 3.5. Multivariable Analysis of Associations with Fat and Protein Content in Bulk-Tank Raw Milk

The multivariable analysis identified a higher number of significant predictors for protein content (*n* = 7) than for fat content (*n* = 3). Among these, two predictors were identified as significant for protein content and another two significant for fat content in both sheep and goat milk (Table 6).

Among the variables included in the multivariable analysis for associations with fat content in the bulk-tank raw milk in sheep flocks (Tables S3 and S4), the following two emerged as significant factors: (a) age of lamb removal from their dams (*p* = 0.016) and (b) breed of ewes (*p* = 0.017). There was also a tendency for significance of the month into the lactation period at sampling (*p* = 0.056).

Among the variables included in the multivariable analysis for associations with protein content in the bulk-tank raw milk in sheep flocks (Tables S3 and S5), the following four emerged as significant factors: (a) somatic cell counts in bulk-tank raw milk (*p* < 0.0001), (b) proportion of *Teladorsagia* larvae in faecal samples (*p* = 0.006), (c) general education of farmers (*p* = 0.008), (d) management system applied in farms (*p* = 0.015), and (e) administration of anthelmintic treatment during the last stage of pregnancy (*p* = 0.016).

Among the variables included in the multivariable analysis for associations with fat content in the bulk-tank raw milk in goat herds (Tables S3 and S6), the following three emerged as significant factors: (a) month into the lactation period at sampling (*p* = 0.017), (b) age of kid removal from their dams (*p* = 0.020), and (c) breed of does (*p* = 0.021).

Among the variables included in the multivariable analysis for associations with protein content in the bulk-tank raw milk in goat herds (Tables S3 and S7), the following four emerged as significant factors: (a) proportion of *Teladorsagia* larvae in faecal samples (*p* = 0.001), (b) somatic cell counts in bulk-tank raw milk (*p* = 0.005), (c) month into the lactation period at sampling (*p* = 0.028), and (d) duration of grazing during the year (*p* = 0.050).

The significant predictors for fat or protein in bulk-tank raw milk are summarised in Table 6 and the detailed results of the multivariable analyses are in Table 7.

### 3.6. Fat and Protein Content in Bulk-Tank Raw Milk Concurrently above the Average Content of All Flocks/Herds in the Study

In this analysis, the desired outcome was achieved in 79 sheep flocks with fat/protein content concurrently over 6.16%/4.43%, respectively. It was also achieved in 32 goat herds with fat/protein content concurrently over 4.77%/3.23%, respectively.

Of these 111 farms, most (30/75, 40.0%) were located in the southern part of the country, a trend that was seen for both sheep and goat farms (19/44 and 11/31 farms, respectively). Less farms were located in the northern part (47/163, 28.8%; 36 sheep and 11 goat farms), in the central part (27/123, 22.0%; 18 sheep and 9 goat farms), and in the islands (7/59, 11.9%; six sheep and one goat farms) (*p* = 0.002 between the geographical parts).

**Figure 4:**
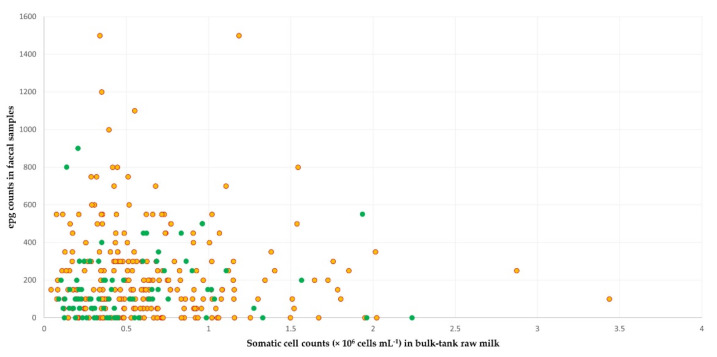
Scatter plot of the somatic cell counts in bulk-tank raw milk and epg counts in faecal samples in sheep flocks: green dots correspond to flocks with fat and protein content in milk concurrently above the average contents of all flocks in the study; yellow–red dots correspond to flocks with fat or protein content below those averages.

In sheep flocks, among the variables included in the multivariable analysis for associations with high fat and protein content concurrently in the bulk-tank raw milk (Tables S3 and S8), the following three emerged as significant factors: (a) somatic cell counts in bulk-tank raw milk (*p* = 0.015), (b) epg counts in faecal samples (*p* = 0.028), and (c) collaboration with a veterinarian (*p* = 0.044) (Table 8). The mean somatic cell counts in the bulk-tank raw milk of flocks that achieved the outcome was 0.411 × 10^6^ cells mL^−1^ (95% CI: 0.349 × 10^6^–0.484 × 10^6^), which was significantly lower (*p* = 0.020) than the somatic cell counts in the flocks that did not achieve the outcome: 0.511 × 10^6^ cells mL^−1^ (95% CI: 0.467 × 10^6^–0.560 × 10^6^). In faecal samples of flocks that achieved the outcome, there was a mean of 167.1 epg (s.e. = 20.1), which was significantly lower (*p* = 0.049) than in the flocks that did not achieve it: 226.0 epg (s.e. = 15.6) (Figure 4, Table S8).

In goat herds, among the variables included in the multivariable analysis for associations with high fat and protein content concurrently in the bulk-tank raw milk (Tables S3 and S9), the following three emerged as significant factors: (a) month into the lactation period at sampling (*p* = 0.007), (b) somatic cell counts in raw milk (*p* = 0.016), and (c) proportion of *Teladorsagia* in faecal samples (*p* = 0.05) (Table 8). The mean somatic cell counts in the bulk-tank raw milk of herds that achieved the outcome was 0.683 × 10^6^ cells mL^−1^ (95% CI: 0.571 × 10^6^–0.819 × 10^6^), which was significantly lower (*p* = 0.015) than the somatic cell counts in the herds that did not achieve it: 0.904 × 10^6^ cells mL^−1^ (95% CI: 0.801 × 10^6^–1.016 × 10^6^) (Table S9).

In sheep, among the 79 flocks with concurrently increased % fat and protein, the proportion of those with concurrently low somatic cell counts (<1.000 × 10^6^ cells mL^−1^) and Trichostrongylidae burdens (≤300 epg in faecal samples) was significantly higher than among the 246 flocks with no concurrently increased % fat and protein: 77% (61/79) versus 63% (155/246) (*p* = 0.020). However, no such significance, but only a tendency, was noted when comparing the same proportion in goat herds: 47% (17/32) versus 36% (31/87), respectively (*p* = 0.08). In contrast, among the 32 goat herds with concurrently increased % fat and protein, the proportion of those with concurrently low somatic cell counts (<1.000 × 10^6^ cells mL^−1^) and low burden of *Teladorsagia* larvae (≤64% in faecal samples) was significantly higher than among the 87 herds with no concurrently increased fat and protein content: 59% (19/32) versus 33% (29/87) (*p* = 0.010).

## 4. Discussion

### 4.1. Preamble

This paper describes a cross-sectional, countrywide, field study in the composition of the bulk-tank raw milk in sheep and goat farms, and this study is one of largest of its type to be reported internationally. Farms from all regions of Greece were included into the study; that way, conditions prevailing throughout the country had been taken into account and factors of regional importance weighed less; moreover, many breeds, some of which have only a regional presence, have thus been included and evaluated in the study.

Many factors were evaluated in the study and, at the end, various predictors for milk composition were identified. Some of these have been discussed extensively in previous publications, e.g., animal breed (which mediates hormone production) [16,17,18,19,27] or animal nutrition (which is associated with nutrient availability) [20,21,28,29]. Nevertheless, some of the factors found to be associated with high fat and/or protein content are difficult to regulate in a farm. For example, the breed of animals is determined at the establishment of the farm, in accord with the general plans and expectations of the farmer. Subsequent changes would be expensive (i.e., requiring the purchase of a large number of animals) or take a long time to implement (i.e., requiring time to modify the genetic background of animals through planned reproduction).

It is noteworthy that, of the 32 frequently cited papers (Table A1) in the topic of milk composition in sheep and goats, not one presented mammary or parasitic infections as risk factors for altered composition of the milk of sheep or goats. Hence, it appears that the potential role of infections in influencing the composition of milk of sheep and goats has not been widely recognised.

This investigation involved a large nationwide survey and looked at many possible risk factors; in these circumstances, it is possible, as is the case with all observational studies, that some of the statistically significant findings were spurious and did not reflect true relationships. Nevertheless, it is noteworthy that, for the main relationships, which are discussed below, statistical significance was evident for both sheep and goats and, in all cases, it was very high (*p* < 0.01; Table 8). Moreover, as discussed below, there is a plausible pathophysiological basis for these relationships and this supports the proposed causal pathways and substantially reduces the potential for spurious statistical findings.

### 4.2. Significance of Increased Somatic Cell Counts in Bulk-Tank Raw Milk

Increased somatic cell counts in bulk-tank milk indicate an increased mastitis incidence in the animals of the farm [30]. Hence, the inverse correlation of somatic cell counts with % protein in the raw milk indirectly reflects the adverse effects of mastitis in the milk composition. Low somatic cell counts also emerged as a significant predictor for combined high fat and protein content in sheep and goats.

Increased somatic cell counts have been associated with adverse effects in the milk composition at an individual animal basis [31,32,33,34]. In contrast, there are only a few studies relating increased somatic cell counts at bulk-tank level with milk composition. The present findings indicate for the first time that somatic cell counts overshadow other factors (e.g., nutritional manipulations) as significant for milk composition. Low somatic cell counts were also found to be important for concurrently high (i.e., above average) % fat and protein.

Changes in the composition of milk (fat, protein) as a result of subclinical mastitis may have a consequent effect in the processing of that milk. In previous studies, attempts have been made to quantify these potential effects. There are, however, large differences in the threshold values of somatic cell counts in milk considered to affect cheese manufacturing ability. Sevi et al. [35] indicated that the renneting ability of milk would decrease with counts over 0.5 × 10^6^ cells mL^−1^, Leitner et al. [36] considered that values over 3.0 × 10^6^ cells mL^−1^ and 6.5 × 10^6^ cells mL^−1^ (sheep and goats, respectively) would affect coagulation of milk, whilst Marti De Olives et al. [34] proposed that cheese manufacturing would be significantly affected only with values over 10.0 × 10^6^ cells mL^−1^. These large differences found in different studies could reflect different procedures followed in the manufacturing of varying cheese types. In ewe milk, Albenzio et al. [37] reported that the impairment of clot firmness could be an outcome of casein breakdown brought about by cathepsin D, which was found to increase when somatic cell counts exceed 1.0 × 10^6^ cells mL^−1^ [38].

It, therefore, becomes evident that measures to reduce somatic cell counts in the bulk-tank milk, which mainly involve the control of mastitis in animals of the farm [39], would benefit the composition of raw milk. Inclusion of a breed with resistance to mastitis will support efforts for mastitis control [40], but it should be noted that, at the breed level, there is an antagonistic genetic correlation between somatic cell counts and milk composition [41,42].

### 4.3. Significance of Parasitic Infections

The identification of parasitic infections as a potential factor influencing milk composition has not been reported previously and it is noteworthy that these findings were seen for both sheep and goats.

The literature is not clear in this aspect. Some studies have not shown an effect of gastrointestinal parasitism on milk composition (sheep: Sechi et al. [43]; goats: Chartier and Hoste [44]). However, Rinaldi et al. [45] indicated that gastrointestinal parasitism could result in up to 30% and 23% less fat and protein content, respectively, in milk. This can be explained by the fact that Trichostrongylidae infections in small ruminants interfere with nutrient digestibility and absorption, leading to a reduction in voluntary feed intake [46,47]. Fatty acids, the precursors for formation of milk fat, are derived from the body and dietary fat. Hence, impaired digestibility and nutrient absorption reduce the body condition score of parasitised animals and also impair the absorption of dietary fat and especially long-chain fatty acids, contributing further to milk fat drop. This reduced digestibility and absorption of dietary fat has an effect on casein synthesis, contributing to low protein content of the milk [48].

It is noteworthy that epg counts were found to be significantly associated with high % fat and protein only in sheep. Moreover, concurrently low epg counts (below 320 epg, which was found to be the threshold for performing anthelmintic treatments in Greece [26]) and somatic cell counts (below 1.000 × 10^6^ cells mL^−1^, which is the threshold above which local dairy companies impose a penalty in raw milk price) were found to be associated with high fat and protein content in sheep, but not in goat milk. This can be a consequence of epg counts from faecal samples more accurately reflecting Trichostrongylidae burdens in sheep than in goats [49,50].

In contrast, the finding of an inverse correlation between the proportion of *Teladorsagia* in faecal samples and the % protein in milk was consistently seen in both sheep and goats. This can possibly be the effect of depressed appetite combined with the losses of plasma protein in a parasitised gastrointestinal tract, as *Teladorsagia* spp. larvae invade the gastric glands in the abomasum and destroy them. This results in impairment of the postabsorptive metabolism of protein and, to a lesser extent, the utilisation of metabolisable energy [51], leading to decreased protein content in milk [47].

Hence, measures to control parasitic infections of animals in a farm would also contribute to high % fat and protein in the bulk-tank raw milk of the farm. The identification of the administration of anthelmintic treatment during the final stage of gestation as a predictor for high protein content (an established scheme that has many advantages [52] and has been widely practiced in sheep farms in Greece) lends further support to this hypothesis.

## 5. Conclusions

Variations in milk composition are the consequences of differences in the relative rates of synthesis and secretion of the various components of milk, which take place at the mammary gland. Thus, many pathways are involved in the milk content produced by sheep and goats. To date, the literature has prioritised non-infection-related parameters (e.g., genetic background, nutrition) to be of importance for the composition of milk.

The results of an extensive countrywide investigation into the fat and protein content of bulk-tank raw milk in 325 flocks and 119 herds have indicated that high somatic cell counts in milk (reflecting the presence of mastitis) and gastrointestinal parasitic infections (mainly *Teladorsagia* infection) appear to exert a more significant influence on fat and protein content of milk, in comparison to non-infection-related factors. The results also indicated that milk fat showed a greater variability than protein, whilst protein was associated with more predictors than fat content.

Therefore, health management in farms should take account of these factors and implement appropriate measures for the control of mastitis and parasitic infections. That way, it will be possible to achieve milk production with high fat and protein content.

## Figures and Tables

**Table 1:** Results (mean ± s.e. ^1^) of fat and protein content (%) of bulk-tank raw milk in 325 sheep flocks and 119 goat herds in Greece.

Parameter	Sheep Milk	Goat Milk
Fat	6.16 ± 0.05	4.77 ± 0.44
	min.: 2.50%, max.: 8.66%	min.: 2.13%, max.: 10.05%
Protein	4.43 ± 0.01	3.23 ± 0.30
	min.: 3.15%, max.: 5.82%	min.: 2.60%, max.: 4.61%

^1^ standard error of the mean.

**Table 2:** Regional results (mean ± s.e. ^1^) of fat and protein content (%) of bulk-tank raw milk in 325 sheep flocks and 119 goat herds in Greece.

Parameter	Sheep Milk					Goat Milk				
	Part of the Country					Part of the Country				
	Central	Islands	North	South	*p*	Central	Islands	North	South	*p*
Fat	6.08 ± 0.06	6.24 ± 0.19	6.12 ± 0.01	6.32 ± 0.10	0.24	4.80 ± 0.19	4.21 ± 0.15	4.78 ± 0.20	5.01 ± 0.26	0.20
Protein	4.50 ± 0.02	4.31 ± 0.03	4.41 ± 0.01	4.38 ± 0.03	<0.001	3.30 ± 0.06	3.01 ± 0.04	3.25 ± 0.05	3.22 ± 0.04	0.013

^1^ standard error of the mean.

**Table 3:** Fat and protein content in bulk-tank raw milk in 325 sheep flocks and 119 goat herds in Greece in accord with proportion (%) of *Teladorsagia* larvae in faecal samples from respective farms.

	Proportion of *Teladorsagia* Larvae in Faecal Samples from Flocks or Herds					
	0% (*n* = 47)	1–63% (*n* = 180)	≥64% (*n* = 98)	0% (*n* = 13)	1–64% (*n* = 66)	≥65% (*n* = 40)
Parameter	Sheep Milk			Goat Milk		
Fat	6.18 ± 0.12	6.16 ± 0.06	6.23 ± 0.09	4.76 ± 0.28	4.93 ± 0.15	4.51 ± 0.20
Protein	4.46 ± 0.05	4.46 ± 0.02	4.35 ± 0.03	3.25 ± 0.06	3.33 ± 0.05	3.05 ± 0.03

**Table 4:** Husbandry-related variables with a significant association (*p* < 0.05) found during univariable analysis with fat and protein content of bulk-tank raw milk in 325 sheep flocks and 119 goat herds in Greece.

Variables	Sheep Milk		Goat Milk	
	Fat Content	Protein Content	Fat Content	Protein Content
Management system applied in the farm		● ^1^		
Month into the lactation period at sampling		●	●	●
Grazing land available to animals	●			
Availability of milking parlour		●	●	
System pressure	●		●	
Breed of ewes/does	●			●
Nutritional modifications performed according to the reproductive stage		●		
Age of lamb/kid removal from their dams			●	
Administration of anthelmintic treatment during the last stage of pregnancy		●		
Duration of grazing during the year				●
Average quantity of hay provided daily to animals during the preceding season		●		
Provision of concentrate feed to adult animals throughout the year	●	●	●	
Type of concentrate feed provided to adult animals	●			

^1^ grey dots indicate a significant association (*p* < 0.05) of the respective variable with fat or protein content of sheep or goat milk.

**Table 5:** Human-resources-related variables with a significant association (*p* < 0.05) found during univariable analysis with fat and protein content of bulk-tank raw milk in 325 sheep flocks and 119 goat herds in Greece.

Variables	Sheep Milk		Goat Milk	
	Fat Content	Protein Content	Fat Content	Protein Content
Length of previous animal farming experience	● ^1^			
General education		●		
Presence of working staff in the farm	●			

^1^ grey dots indicate a significant association (*p* < 0.05) of the respective variable with fat or protein content of sheep or goat milk.

**Table 6:** Significance of associations, as found during multivariable analyses, of variables with fat and protein content of bulk-tank raw milk in 325 sheep flocks and 119 goat herds in Greece.

Variables	Sheep Milk		Goat Milk	
	Fat Content (*p* Values)	Protein Content (*p* Values)	Fat Content (*p* Values)	Protein Content (*p* Values)
Somatic cell counts in bulk-tank raw milk		<0.0001		0.005
Proportion of *Teladorsagia* larvae in faecal samples		0.006		0.001
Management system applied in the farm		0.015		
Month into the lactation period at sampling	(0.056)		0.017	0.028
Breed of ewes/does	0.017		0.021	
Age of lamb/kid removal from their dams	0.016		0.020	
Administration of anthelmintic treatment during the last stage of pregnancy		0.016		
Duration of grazing during the year				0.050
General education of the farmer		0.008		

**Table 7:** Detailed results of multivariable analyses for associations with fat and protein content in bulk-tank raw milk of 325 sheep flocks and 119 goat herds in Greece.

Variables	Regression Coefficients (±Standard Error)	*p*
Fat content in bulk-tank raw milk of sheep flocks		
Age of lamb removal from their dams		0.016
<45 days	0.358 ± 0.159	0.025
45–60 days	0.331 ± 0.154	0.032
>60 days	reference	-
Breed of ewes		0.017
Assaf	0.940 ± 0.822	0.25
Awassi	0.305 ± 1.144	0.79
Boutsko	1.430 ± 0.991	0.15
Chios	0.609 ± 0.819	0.46
Crossbreed	1.035 ± 0.818	0.21
Friesarta	0.765 ± 0.842	0.36
Friesian	1.291 ± 0.840	0.13
Karagouniko	0.505 ± 0.886	0.57
Kefallinia	reference	-
Lacaune	1.179 ± 0.813	0.15
Local	1.080 ± 0.816	0.19
Mytilini	1.766 ± 0.831	0.034
Sfakia	0.663 ± 0.874	0.45
Protein content in bulk-tank raw milk of sheep flocks		
Somatic cell counts in bulk-tank raw milk		<0.001
per unit ^1^ change	–0.052 ± 0.013	<0.001
Proportion of *Teladorsagia* larvaein faecal samples		0.006
0%	reference	-
1–63%	–0.004 ± 0.410	0.92
≥64%	–0.114 ± 0.045	0.012
General education of the farmer		0.008
Primary education	0.124 ± 0.038	0.001
Secondary and post-secondary education	reference	-
Tertiary education	0.043 ± 0.042	0.31
Management system applied in farms		0.015
Intensive	0.141 ± 0.063	0.027
Semi-intensive	0.149 ± 0.055	0.007
Semi-extensive	0.065 ± 0.056	0.25
Extensive	reference	-
Administration of anthelmintic treatment during the last stage of pregnancy		0.016
Yes	0.078 ± 0.031	0.012
No	reference	-
Fat content in bulk-tank raw milk of goat herds		
Month into the lactation period at sampling		0.017
0–1st	1.476 ± 0.595	0.015
2nd–5th	0.991 ± 0.88	0.029
6th–9th	0.642 ± 0.458	0.16
After 9th	reference	-
Age of kid removal from their dams		0.020
<45 days	reference	-
45–60 days	0.157 ± 0.295	0.60
>60 days	0.676 ± 0.289	0.021
Breed of does		0.021
Alpine	reference	-
Crossbreed	0.636 ± 0.493	0.20
Damascus	0.863 ± 0.493	0.08
Kefallinia	1.814 ± 1.274	0.16
Local (*Capra prisca*)	0.999 ± 0.438	0.024
Murcia	1.184 ± 0.524	0.026
Saanen	0.498 ± 0.674	0.46
Skopelos	1.337 ± 0.674	0.050
Protein content in bulk-tank raw milk of goat herds		
Proportion of *Teladorsagia* larvae in faecal samples		0.001
0%	–0.079 ± 0.085	0.35
1–64%	reference	-
≥65%	–0.281 ± 0.056	<0.001
Somatic cell counts in bulk-tank raw milk		0.005
per unit ^1^ change	–0.106 ± 0.033	0.002
Month into the lactation period at sampling		0.028
0–1st (*n* = 23)	0.304 ± 0.120	0.012
2nd–5th (*n* = 138)	0.176 ± 0.067	0.010
6th–9th (*n* = 147)	reference	-
After 9th (*n* = 17)	0.082 ± 0.120	0.50
Duration of grazing during the year		0.050
No grazing	0.088 ± 0.111	0.43
2–5 months	0.295± 0.090	0.001
6–10 months	0.030 ± 0.063	0.64
10–11 months	reference	-

^1^ ascending units: 25, 50, 100, 200, 400, 800, 1600, 3200, 6400, 12,800, 25,600, etc., cells mL^−1^.

**Table 8:** Detailed results of multivariable analysis for associations with high fat and protein content concurrently in bulk-tank raw milk of 325 sheep flocks and 119 goat herds in Greece.

Variables	Odds Ratios ^1^ (95% Confidence Intervals)	*p*
Sheep flocks		
Somatic cell counts		0.015
per unit ^2^ increase	0.946 (0.925–0.968)	0.014
epg counts in faecal samples		0.028
≤300 epg (69/259, 26.6% of flocks)	2.034 (0.983–4.208)	0.06
≥350 epg (10/66, 15.2% of flocks)	reference	-
Collaboration with a veterinarian		0.044
Yes (73/277, 26.4%)	2.505 (1.022–6.138)	0.045
No (6/48, 12.5%)	reference	0.045
Goat herds		
Month into the lactation period		0.007
0–1st month (6/8, 75.0% of herds)	21.000 (1.504–293.268)	0.024
2nd–5th month (18/60, 30.0% of herds)	3.000 (0.344–26.193)	0.32
6th–9th month (7/43, 16.3% of herds)	1.361 (0.144–12.866)	0.79
After 9th month (1/8, 12.5% of herds)	reference	-
Somatic cell counts		0.016
per unit ^2^ increase	0.893 (0.849–0.937)	0.015
Proportion of *Teladorsagia* larvae in faecal samples		0.050
0% (4/13, 30.8% of herds)	4.000 (0.835–19.162)	0.08
1–64% (24/66, 36.4% of herds)	5.143 (1.631–16.215)	0.005
≥65% (4/40, 10.0% of herds)	reference	-

^1^ odds ratio for bulk-tank raw milk being concurrently high for fat and protein. ^2^ ascending units: 25, 50, 100, 200, 400, 800, 1600, 3200, 6400, 12,800, 25,600, etc., cells mL^−1^.

## Data Availability

Most data presented in this study are in the Supplementary Materials. The remaining data are available on request from the corresponding author. The data are not publicly available as they form part of the PhD thesis of the first author, which has not yet been examined, approved, and uploaded in the official depository of PhD theses from Greek Universities.

## Supplementary Materials

The following are available online at https://www.mdpi.com/article/10.3390/foods11030443/s1, Table S1: Husbandry-related variables (*n* = 37) evaluated for potential association with fat and protein content in the bulk-tank raw milk of 325 sheep and 119 goat farms during a countrywide investigation in Greece, Table S2: Human resources-related variables (*n* = 6) evaluated for potential association with fat and protein content in the bulk-tank raw milk of 325 sheep and 119 goat farms during a countrywide investigation in Greece, Table S3: Details of multivariable models employed for the evaluation of the fat and protein content in the bulk-tank raw milk of 325 sheep and 119 goat farms during a countrywide investigation in Greece, Table S4: Effects of husbandry- and human resources-related factors (*n* = 43) in the fat content (%) in the bulk-tank raw milk of 325 sheep flocks in Greece, Table S5: Effects of husbandry- and human resources-related factors (*n* = 43) in the protein content (%) in the bulk-tank raw milk of 325 sheep flocks in Greece, Table S6: Effects of husbandry- and human resources-related factors (*n* = 43) in the fat content (%) in the bulk-tank raw milk of 119 goat herds in Greece, Table S7: Effects of husbandry- and human resources-related factors (*n* = 43) in the fat content (%) in the bulk-tank raw milk of 119 goat herds in Greece, Table S8: Results of univariable analysis of variables (*n* = 32) for evaluation of the outcome “fat and protein content in bulk-tank milk concurrently above the average contents found for all flocks” in 325 sheep flocks in Greece, Table S9: Results of univariable analysis of variables (*n* = 19) for evaluation of the outcome “fat and protein content in bulk-tank milk concurrently above the average contents found for all flocks” in 119 goat herds in Greece.

## Appendix A

**Table A1:** List ^1^ of the 32 more frequently cited ^2^ relevant papers (research articles or reviews) published from 1970 until the end of 2021, found in the platform Web of Science by using the search terms [*milk*] AND [*composition* OR *content*] AND [*sheep* OR *goat**].

Authors	Title of Paper	Year of Publication	Origin of Paper ^3^	Bibliographical Details
Jenness, R.	Composition and characteristics of goat milk—review 1968–1979	1980	USA	*J. Dairy Sci.* 63, 1605–1630
Barry, T.N.; McNabb, W.C.	The implications of condensed tannins on the nutritive value of temperate forages fed to ruminants	1999	New Zealand	*Br. J. Nutr.* 81, 263–272
Martin, P. et al.	The impact of genetic polymorphisms on the protein composition of ruminant milks	2002	France, Poland	*Reprod. Nutr. Dev.* 42, 433–459
Chilliard, Y. et al.	A review of nutritional and physiological factors affecting goat milk lipid synthesis and lipolysis	2003	France	*J. Dairy Sci.* 86, 1751–1770
Chilliard, Y.; Ferlay, A.	Dietary lipids and forages interactions on cow and goat milk fatty acid composition and sensory properties	2004	France	*Reprod. Nutr. Dev.* 44, 467–492
Haenlein, G.F.W.	Goat milk in human nutrition	2004	USA	*Small Rumin. Res.* 51, 155–163
Chilliard, Y. et al.	Diet, rumen biohydrogenation and nutritional quality of cow and goat milk fat	2007	France	*Eur. J. Lipid Sci. Technol.* 109, 828–855
Morand-Fehr, P. et al.	Influence of farming and feeding systems on composition and quality of goat and sheep milk	2007	France, Italy	*Small Rumin. Res.* 68, 20–34
Park, Y. et al.	Physico-chemical characteristics of goat and sheep milk	2007	Spain, USA	*Small Rumin. Res.* 68, 88–113
Sampelayo, M.R.S. et al.	Influence of type of diet on the fat constituents of goat and sheep milk	2007	France, Spain	*Small Rumin. Res.* 68, 42–63
Bernard, L. et al.	Expression and nutritional regulation of lipogenic genes in the ruminant lactating mammary gland	2008	France	*Adv. Exp. Med. Biol.* 606, 67–108
Jenkins, T.C. et al.	Recent advances in biohydrogenation of unsaturated fatty acids within the rumen microbial ecosystem	2008	UK, USA	*J. Anim. Sci.* 86, 397–412
Molina-Alcaide, E.; Yanez-Ruiz, D.R.	Potential use of olive by-products in ruminant feeding: a review	2008	Spain, UK	*Anim. Feed Sci. Technol.* 147, 247–264
Raynal-Ljutovac, K. et al.	Composition of goat and sheep milk products: an update	2008	France	*Small Rumin. Res.* 79, 57–72
Vasta, V. et al.	Alternative feed resources and their effects on the quality of meat and milk from small ruminant	2008	Italy	*Anim. Feed Sci. Technol.* 147, 223–246
Ceballos, L.S. et al.	Composition of goat and cow milk produced under similar conditions and analyzed by identical methodology	2009	Spain	*J. Food Comp. Anal.* 22, 322–329
Shingfield, K.J. et al.	Role of trans fatty acids in the nutritional regulation of mammary lipogenesis in ruminants	2009	Finland	*Animal* 4, 1140–1166
Vasta, V. et al.	Metabolic fate of fatty acids involved in ruminal biohydrogenation in sheep fed concentrate or herbage with or without tannins	2009	Italy	*J. Anim. Sci.* 87, 2674–2684
Silanikove, N. et al.	Recent advances in exploiting goat’s milk: quality, safety and production aspects	2010	Israel, New Zealand	*Small Rumin. Res.* 89, 110–124
Barlowska, J. et al.	Nutritional value and technological suitability of milk from various animal species used for dairy production	2011	Poland	*Compr. Rev. Food Sci. Food Safety* 10, 291–302
Grainger, C.; Beauchemin, K.A.	Can enteric methane emissions from ruminants be lowered without lowering their production?	2011	Canada	*Anim. Feed Sci. Technol.* 166–167, 308–320
Tannock, G.W. et al.	Comparison of the compositions of the stool microbiotas of infants fed goat milk formula, cow milk-based formula, or breast milk	2013	Australia, New Zealand	*Appl. Env. Microbiol.* 79, 3040–3048
Zou, X.Q. et al.	Lipid composition analysis of milk fats from different mammalian species: potential for use as human milk fat substitutes	2013	Denmark, PRC	*J. Agric. Food Chem.* 61, 7070–7080
Claeys, W.L. et al.	Consumption of raw or heated milk from different species: An evaluation of the nutritional and potential health benefits	2014	Belgium	*Food Contr.* 42, 188–201
Nudda, A. et al.	Feeding strategies to design the fatty acid profile of sheep milk and cheese	2014	Italy	*Rev. Bras. Zoot.* 43, 445–456
Buccioni, A. et al.	Milk fatty acid composition, rumen microbial population, and animal performances in response to diets rich in linoleic acid supplemented with chestnut or quebracho tannins in dairy ewes	2015	Italy	*J. Dairy Sci.* 98, 1145–1156
Rezaei, R. et al.	Amino acids and mammary gland development: nutritional implications for milk production and neonatal growth	2016	PRC, USA	*J. Anim. Sci. Biotechnol.* 7, 20
Xu, H.F. et al.	Overexpression of SREBP1 (sterol regulatory element binding protein 1) promotes de novo fatty acid synthesis and triacylglycerol accumulation in goat mammary epithelial cells	2016	PRC	*J. Dairy Sci.* 99, 783–795
Balthazar, C.F. et al.	Sheep milk: physicochemical characteristics and relevance for functional food development	2017	Brazil, Italy	*Compr. Rev. Food Sci. Food Safety* 16, 247–262
Clark, S; Garcia, M.B.M.	Advances in goat milk research	2017	USA	*J. Dairy Sci.* 100, 10026–10044
Goldansaz, S.A. et al.	Livestock metabolomics and the livestock metabolome: a systematic review	2017	Canada	*Plos One* 12, e0177675
Li, Q.Q. et al.	Lipidomics profiling of goat milk, soymilk and bovine milk by UPLC-Q-Exactive Orbitrap Mass Spectrometry	2017	PRC, New Zealand	*Food Chem.* 224, 302–309

^1^ listed papers presented in chronological and alphabetical (according to surname of the first author) order. ^2^ >10.0 citations per year after publication. ^3^ PRC: People’s Republic of China, UK: United Kingdom, USA: United States of America.
