# Supplementary material for: High Milk Somatic Cell Counts and Increased Teladorsagia Burdens Overshadow Non-Infection-Related Factors as Predictors of Fat and Protein Content of Bulk-Tank Raw Milk in Sheep and Goat Farms
Source: Foods. 2022 Feb 2;11(3):443. doi: 10.3390/foods11030443 (PMC8834117; doi:10.3390/foods11030443)
Supplement: Supplementary file 1 [file foods-11-00443-s001.zip › foods-1543755-supplementary.pdf]

High Milk Somatic Cell Counts and Increased Teladorsagia Burdens Overshadow Non-Infection Related Factors as Predictors of Fat and Protein Content of Bulk-Tank Raw Milk in Sheep and Goat Farms

Daphne T. Lianou, Charalambia K. Michael, Dimitris A. Gougoulis, Peter J. Cripps, Natalia G.C. Vasileiou, Nikolaos Solomakos, Efthymia Petinaki, Angeliki I. Katsafadou, Elisavet Angelidou, Konstantinos Arsenopoulos, Elias Papadopoulos, Marzia Albenzio, Vasia S. Mavrogianni, Mariangela Caroprese and George C. Fthenakis

Table S1. Husbandry-related variables (n = 37) evaluated for potential association with fat and protein content in the bulk-tank raw milk of 325 sheep and 119 goat farms during a countrywide investigation in Greece.

Management system applied in the farm (description according to EFSA classification [1])
Month into the lactation period at sampling (month)
Availability of straw bedding (yes / no)
Availability of mechanical ventilators (yes / no)
Grazing practiced (yes / no)
Grazing land available to animals (acres per animal)
Availability of milking parlour (yes / no)
Number of milking units in the parlour (no.)
Number of available milking units per animal position (no.)
System pressure (kPa.)
Type of flow line (low / high / other)
No. of female animals in the farm (no.)
Breed of ewes /does (description)
Average age of culling female animals (years)
Total milk quantity per ewe / doe obtained during the preceding milking period (litres)
Average number of lambs / kids born per ewe / doe (no.)
Collaboration with a veterinarian (yes / no)
Clinical mastitis annual incidence risk in the flock / herd (%)
Application of reproductive control practices in the farm (yes / no)
Nutritional modifications performed according to the reproductive stage (yes / no)
Method for drying-off at the end of the lactation period (abrupt / progressive)
Age of lamb / kid removal from their dams (days)
Daily number of milking sessions (no.)
Number of feet care sessions provided annually to the female animals annually (no.)
Shearing of animals (yes / no)
Vaccination against mastitis (yes / no)
Vaccination against contagious agalactia (yes / no)
Administration of anthelmintic treatment during the last stage of pregnancy (yes / no)
Duration of grazing during the year (no. of months)

Provision of hay as fodder to animals (yes / no)
Average quantity of hay provided daily to animals during the preceding season (kg)
Provision of straw to animals (yes / no)
Provision of silage to adult animals (yes / no)
Provision of finished feed (concentrate) to adult animals (yes / no)
Provision of finished feed (concentrate) to adult animals throughout the year (yes / no)
Type of finished feed (concentrate) provided to adult animals (description)
Average quantity of finished feed (concentrate) provided daily to animals during the preceding season (kg)

[1] management system classified as intensive, semi-intensive, semi-extensive, extensive (European Food Safety Authority. Scientific opinion on the welfare risks related to the farming of sheep for wool, meat and milk production. *EFSA J.* **2014**, *12*, 3933–4060.).

Table S2. Human resources-related variables (*n* = 6) evaluated for potential association with fat and protein content in the bulk-tank raw milk of 325 sheep and 119 goat farms during a countrywide investigation in Greece.

Age of farmer (years)
Length of previous animal farming experience (years)
General education (description)
Farmer by profession (yes / no)
Family tradition in farming (yes / no)
Presence of working staff in the farm (yes/no)

Table S3. Details of multivariable models employed for the evaluation of the fat and protein content in the bulk-tank raw milk of 325 sheep and 119 goat farms during a countrywide investigation in Greece.

**Outcome**	**Variables offered to the multivariable models (*n*)**	**Variables required in the final models**
Fat content – sheep milk	16	(a) Month into the lactation period at sampling, (b) Breed of ewes, (c) Age of lamb removal from their dams, (d) Provision of finished feed (concentrate) to adult animals throughout the year, (e) Length of previous animal farming experience of the farmer, (f) presence of working staff in the farm
Protein content – sheep milk	21	(a) Somatic cell counts in bulk tank milk, (b) Proportion of *Teladorsagia* larvae in faecal samples, (c) Management system applied in the farm, (d) Month into the lactation period at sampling, (e) Administration of anthelmintic treatment during the last stage of pregnancy, (f) Provision of finished feed (concentrate) to adult animals throughout the year, (g) General education of farmer
Fat content – goat milk	11	(a) Somatic cell counts in bulk tank milk, (b) Total bacterial counts in bulk-tank milk, (c) Month into the lactation period at sampling, (d) Breed of does, (e) Collaboration with a veterinarian, (f) Age of kid removal from their dams, (g) General education of farmer
Protein content – goat milk	8	(a) Somatic cell counts in bulk tank milk, (b) Proportion of *Teladorsagia* larvae in faecal samples, (c) Month into the lactation period at sampling, (d) Duration of grazing during the year
Fat and protein content concurrently above the average contents of all flocks - sheep	8	(a) Somatic cell counts in bulk tank milk, (b) epg counts in faecal samples, (c) Collaboration with a veterinarian, (d) General education of the farmer
Fat and protein content concurrently above the average contents of all herds - goats	5	(a) Somatic cell counts in bulk tank milk, (b) Proportion of *Teladorsagia* larvae in faecal samples, (c) Month into the lactation period at sampling, (d) Breed of does

Table S4. Effects of husbandry- and human resources-related factors (*n* = 43) in the fat content (%) in the bulk-tank raw milk of 325 sheep flocks in Greece.

**Management system applied in the farm**				
Intensive (n = 43)	Semi-intensive (n = 151)	Semi-extensive (n = 107)	Extensive (n = 24)	*p*
6.03 ± 0.09	6.24 ± 0.06	6.11 ± 0.09	6.16 ± 0.23	0.44
**Month into the lactation period at sampling**				
0 – 1st (n = 23)	2nd – 5th (n = 138)	6th – 9th (n = 147)	After 9th (n = 17)	*p*
5.77 ± 0.13	6.13 ± 0.07	6.25 ± 0.07	6.23 ± 0.33	0.07
**Availability of straw bedding**				
Yes (n = 268)	No (n = 57)			*p*
6.14 ± 0.05	6.28 ± 0.12			0.24
**Availability of mechanical ventilators**				
Yes (n = 47)	No (n = 278)			*p*
6.12 ± 0.12	6.17 ± 0.05			0.67
**Grazing practiced**				
Yes (n = 281)	No (n = 44)			*p*
6.19 ± 0.05	6.00 ± 0.09			0.15
**Grazing land available to animals**				
≤ 0.50 ac. per animal (n = 118)	0.51-2.00 ac. per animal (n = 130)	> 2.00 ac. per animal (n = 77)		*P*
6.03 ± 0.06	6.32 ± 0.07	6.10 ± 0.12		0.022
**Availability of milking parlour**				
Yes (n = 255)	No (n = 70)			*p*
6.15 ± 0.05	6.22 ± 0.11			0.53
**Number of milking units in the parlour**				
< 24 (n = 174)	24 (n = 65)	> 24 (n = 16)		*p*
6.18 ± 0.06	6.14 ± 0.10	5.86 ± 0.18		0.33
**Number of available milking units per animal position**				
< 1 (n = 177)	1 (n = 78)			*p*
6.21 ± 0.06	6.01 ± 0.09			0.08
**System pressure**				
< 38 kPa (n = 22)	38 - 42 kPa (n = 203)	> 42 kPa (n = 30)		*p*
6.05 ± 0.24	6.21 ± 0.06	5.79 ± 0.15		0.029
**Type of flow line**				
High (n = 182)	Low (n = 55)	Other (n = 18)		*p*
6.18 ± 0.06	6.12 ± 0.12	5.94 ± 0.19		0.48
**No. of ewes in the flock**				
≤ 165 ewes (n = 88)	166 - 330 ewes (n = 120)	331 - 500 ewes (n = 66)	> 500 ewes (n = 51)	*p*
6.02 ± 0.10	6.28 ± 0.07	6.22 ± 0.11	6.08 ± 0.11	0.13
**Breed of ewes**				
Assaf (n = 30)	Awassi (n = 1)	Boutsko (n = 2)	Chios (n = 44)	*p*
6.06 ± 0.12	5.43	6.55 ± 1.14	5.73 ± 0.15	< 0.0001
Crossbreeds (n = 43)	Friesarta (n = 12)	Friesian (n = 13)	Karagouniko (n = 5)	
6.16 ± 0.11	5.89 ± 0.15	6.41 ± 0.22	5.63 ± 0.62	

Kefallinia (n = 1)	Lacaune (n = 95)	Local (n = 55)	Mytilini (n = 18)	
5.12	6.30 ± 0.08	6.20 ± 0.12	6.89 ± 0.15	
Sfakia (n = 6)				
5.78 ± 0.23				
**Average age of culling ewes**				
≤ 6 years (n = 226)		> 6 years (n = 99)		*p*
6.17 ± 0.06		6.16 ± 0.08		0.92
**Total milk quantity per ewe obtained during the preceding milking period**				
≤ 200 L (n = 174)	201 - 400 L (n = 140)	> 400 L (n = 11)		*p*
6.18 ± 0.07	6.13 ± 0.07	6.33 ± 0.18		0.61
**Average number of lambs born per ewe**				
≤ 1.50 (n = 280)		> 1.50 (n = 45)		*p*
6.16 ± 0.05		6.20 ± 0.13		0.73
**Collaboration with a veterinarian**				
Yes (n = 277)		No (n = 48)		*p*
6.17 ± 0.05		6.10 ± 0.14		0.59
**Clinical mastitis annual incidence risk in the flock**				
≤ 0.50% (n = 269)		> 0.50% (n = 56)		*p*
6.15 ± 0.10		6.15 ± 0.05		0.91
**Application of reproductive control practices in the farm**				
Yes (n = 100)		No (n = 225)		*p*
6.11 ± 0.08		6.19 ± 0.06		0.38
**Nutritional modifications performed according to the reproductive stage**				
Yes (n = 229)		No (n = 96)		*p*
6.16 ± 0.05		6.17 ± 0.09		0.90
**Method for drying-off at the end of the lactation period**				
Abrupt (n = 12)		Progressive (n = 313)		*p*
6.25 ± 0.20		6.16 ± 0.05		0.70
**Age of lamb removal from their dams**				
< 45 days (n = 119)	45 – 60 days (n = 170)	> 60 days (n = 36)		*p*
6.22 ± 0.07	6.19 ± 0.07	5.86 ± 0.17		0.07
**Daily number of milking sessions**				
One (n = 1)	Two (n = 264)	Three (n = 60)		*p*
6.85	6.17 ± 0.05	6.13 ± 0.09		0.75
**Number of feet care sessions provided to the ewes annually**				
No feet care provided (n = 102)	1 - 2 (n = 203)	> 2 (n = 20)		*p*
6.22 ± 0.09	6.10 ± 0.06	6.52 ± 0.14		0.07
**Shearing of animals**				
Yes (n = 319)		No (n = 6)		*p*
6.16 ± 0.05		6.31 ± 0.41		0.68
**Vaccination against mastitis**				
Yes (n = 126)		No (n = 199)		*p*
6.18 ± 0.07		6.15 ± 0.06		0.77

**Vaccination against contagious agalactia**					
Yes (n = 186)	No (n = 139)				*p*
6.16 ± 0.06	6.17 ± 0.08				0.97
**Administration of anthelmintic treatment during the last stage of pregnancy**					
Yes (n = 224)	No (n = 101)				*p*
6.20 ± 0.05	6.08 ± 0.09				0.23
**Duration of grazing during the year**					
No grazing (n = 44)	1 - 5 months (n = 46)	6 - 10 months (n = 124)	11 - 12 months (n = 111)		*p*
6.11 ± 0.09	6.06 ± 0.13	6.21 ± 0.07	6.17 ± 0.09		0.72
**Provision of hay as fodder to animals**					
Yes (n = 324)	No (n = 1)				*p*
6.16 ± 0.05	6.63				0.44
**Average quantity of hay provided daily to animals during the preceding season**					
≤ 0.6 kg (n = 109)	> 0.6 kg (n = 216)				*p*
6.26 ± 0.09	6.11 ± 0.05				0.13
**Provision of straw to animals**					
Yes (n = 258)	No (n = 67)				*p*
6.18 ± 0.05	6.11 ± 0.14				0.57
**Provision of silage to adult animals**					
Yes (n = 72)	No (n = 253)				*p*
6.18 ± 0.09	6.16 ± 0.05				0.90
**Provision of finished feed (concentrate) to adult animals**					
Yes (n = 321)	No (n = 4)				*p*
6.17 ± 0.05	6.02 ± 0.63				0.73
**Provision of finished feed (concentrate) to adult animals throughout the year**					
Yes (n = 219)	No (n = 106)				*p*
6.09 ± 0.05	6.34 ± 0.09				0.012
**Type of finished feed (concentrate) to adult animals**					
Mash (n = 122)	Pelleted (n = 101)	Flakes (n = 4)	Crumbled (n = 87)	Other (n = 7)	*p*
6.09 ± 0.07	6.08 ± 0.08	6.45 ± 0.38	6.38 ± 0.09	5.83 ± 0.39	0.05
**Average quantity of finished feed (concentrate) provided daily to animals during the preceding season**					
≤ 0.6 kg (n = 140)	0.61 – 1.2 kg (n = 148)	> 1.2 kg (n = 37)			*p*
6.22 ± 0.07	6.10 ± 0.07	6.20 ± 0.12			0.42
**Age of farmer**					
Up to 50 years (n = 197)	Over 50 years (n = 128)				*p*
6.18 ± 0.06	6.14 ± 0.07				0.74
**Length of previous animal farming experience**					
≤ 5 years (n = 74)	> 5 years (n = 251)				*p*
5.94 ± 0.10	6.23 ± 0.05				0.008
**General education of farmer**					
Primary (n = 57)	Secondary and post-secondary (n = 225)	Tertiary (n = 43)			*p*
6.17 ± 0.10	6.15 ± 0.06	6.21 ± 0.13			0.92

Farmer by profession		
Yes (n = 292)	No (n = 33)	*p*
6.18 ± 0.05	6.03 ± 0.14	0.33
**Family tradition in farming**		
Yes (n = 283)	No (n = 42)	*p*
6.19 ± 0.05	5.98 ± 0.11	0.13
**Presence of working staff in the farm**		
Yes (n = 123)	No (n = 202)	*p*
6.01 ± 0.07	6.26 ± 0.06	0.008

Table S5. Effects of husbandry- and human resources-related factors (*n* = 43) in the protein content (%) in the bulk-tank raw milk of 325 sheep flocks in Greece.

**Management system applied in the farm**				
Intensive (n = 43)	Semi-intensive (n = 151)	Semi-extensive (n = 107)	Extensive (n = 24)	*p*
4.46 ± 0.02	4.47 ± 0.02	4.38 ± 0.02	4.32 ± 0.04	0.021
**Month into the lactation period at sampling**				
0 – 1st (n = 23)	2nd – 5th (n = 138)	6th – 9th (n = 147)	After 9th (n = 17)	*p*
4.42 ± 0.07	4.47 ± 0.02	4.40 ± 0.02	4.33 ± 0.06	0.045
**Availability of straw bedding**				
Yes (n = 268)	No (n = 57)			*p*
4.44 ± 0.02	4.37 ± 0.04			0.09
**Availability of mechanical ventilators**				
Yes (n = 47)	No (n = 278)			*p*
4.45 ± 0.03	4.42 ± 0.02			0.53
**Grazing practiced**				
Yes (n = 281)	No (n = 44)			*p*
4.42 ± 0.02	4.47 ± 0.02			0.91
**Grazing land available to animals**				
≤ 0.50 ac. per animal (n = 118)	0.51-2.00 ac. per animal (n = 130)	> 2.00 ac. per animal (n = 77)		*p*
4.46 ± 0.02	4.42 ± 0.02	4.38 ± 0.03		0.09
**Availability of milking parlour**				
Yes (n = 255)	No (n = 70)			*p*
4.45 ± 0.02	4.35 ± 0.03			0.005
**Number of milking units in the parlour**				
< 24 (n = 174)	24 (n = 65)	> 24 (n = 16)		*p*
4.46 ± 0.02	4.40 ± 0.03	4.45 ± 0.06		0.20
**Number of available milking units per animal position**				
< 1 (n = 177)	1 (n = 78)			*p*
4.45 ± 0.02	4.43 ± 0.03			0.58
**System pressure**				
< 38 kPa (n = 22)	38 - 42 kPa (n = 203)	> 42 kPa (n = 30)		*p*
4.44 ± 0.07	4.44 ± 0.02	4.48 ± 0.04		0.76
**Type of flow line**				
High (n = 182)	Low (n = 55)	Other (n = 18)		*p*
4.45 ± 0.02	4.41 ± 0.03	4.54 ± 0.04		0.14
**No. of ewes in the flock**				
≤ 165 ewes (n = 88)	166 - 330 ewes (n = 120)	331 - 500 ewes (n = 66)	> 500 ewes (n = 51)	*p*
4.42 ± 0.03	4.45 ± 0.03	4.40 ± 0.02	4.42 ± 0.04	0.61
**Breed of ewes**				
Assaf (n = 30)	Awassi (n = 1)	Boutsko (n = 2)	Chios (n = 44)	*p*
4.36 ± 0.03	4.73	4.60 ± 0.05	4.47 ± 0.04	0.24
Crossbreeds (n = 43)	Friesarta (n = 12)	Friesian (n = 13)	Karagouniko (n = 5)	
4.42 ± 0.06	4.51 ± 0.06	4.37 ± 0.09	4.35 ± 0.11	

Kefallinia (n = 1)	Lacaune (n = 95)	Local (n = 55)	Mytilini (n = 18)	
4.55	4.47 ± 0.03	4.39 ± 0.03	4.35 ± 0.03	
Sfakia (n = 6)				
4.35 ± 0.04				
**Average age of culling ewes**				
≤ 6 years (n = 226)		> 6 years (n = 99)		*p*
4.43 ± 0.02		4.42 ± 0.03		0.84
**Total milk quantity per ewe obtained during the preceding milking period**				
≤ 200 L (n = 174)	201 - 400 L (n = 140)	> 400 L (n = 11)		*p*
4.43 ± 0.02	4.42 ± 0.02	4.41 ± 0.05		0.73
**Average number of lambs born per ewe**				
≤ 1.50 (n = 280)		> 1.50 (n = 45)		*p*
4.42 ± 0.02		4.48 ± 0.03		0.14
**Collaboration with a veterinarian**				
Yes (n = 277)		No (n = 48)		*p*
4.44 ± 0.02		4.37 ± 0.04		0.09
**Clinical mastitis annual incidence risk in the flock**				
≤ 0.50% (n = 269)		> 0.50% (n = 56)		*p*
4.41 ± 0.03		4.43 ± 0.02		0.59
**Application of reproductive control practices in the farm**				
Yes (n = 100)		No (n = 225)		*p*
4.45 ± 0.02		4.42 ± 0.02		0.26
**Nutritional modifications performed according to the reproductive stage**				
Yes (n = 229)		No (n = 96)		*p*
4.44 ± 0.02		4.38 ± 0.03		0.05
**Method for drying-off at the end of the lactation period**				
Abrupt (n = 12)		Progressive (n = 313)		*p*
4.47 ± 0.04		4.42 ± 0.01		0.59
**Age of lamb removal from their dams**				
< 45 days (n = 119)	45 – 60 days (n = 170)	> 60 days (n = 36)		*p*
4.46 ± 0.02	4.41 ± 0.02	4.37 ± 0.06		0.12
**Daily number of milking sessions**				
One (n = 1)	Two (n = 264)	Three (n = 60)		*p*
4.45	4.42 ± 0.02	4.47 ± 0.03		0.15
**Number of feet care sessions provided to the ewes annually**				
No feet care provided (n = 102)	1 - 2 (n = 203)	> 2 (n = 20)		*p*
4.39 ± 0.02	4.44 ± 0.02	4.44 ± 0.03		0.16
**Shearing of animals**				
Yes (n = 319)		No (n = 6)		*p*
4.42 ± 0.01		4.55 ± 0.14		0.25
**Vaccination against mastitis**				
Yes (n = 126)		No (n = 199)		*p*
4.43 ± 0.02		4.42 ± 0.02		0.70

**Vaccination against contagious agalactia**					
Yes (n = 186)	No (n = 139)				*p*
4.44 ± 0.02	4.41 ± 0.02				0.43
**Administration of anthelmintic treatment during the last stage of pregnancy**					
Yes (n = 224)	No (n = 101)				*p*
4.45 ± 0.02	4.38 ± 0.03				0.019
**Duration of grazing during the year**					
No grazing (n = 44)	1 - 5 months (n = 46)	6 - 10 months (n = 124)	11 - 12 months (n = 111)		*p*
4.47 ± 0.02	4.46 ± 0.04	4.44 ± 0.02	4.38 ± 0.03		0.07
**Provision of hay as fodder to animals**					
Yes (n = 324)	No (n = 1)				*p*
4.43 ± 0.01	4.34				0.62
**Average quantity of hay provided daily to animals during the preceding season**					
≤ 0.6 kg (n = 109)	> 0.6 kg (n = 216)				*p*
4.38 ± 0.02	4.45 ± 0.02				0.030
**Provision of straw to animals**					
Yes (n = 258)	No (n = 67)				*p*
4.43 ± 0.02	4.40 ± 0.03				0.41
**Provision of silage to adult animals**					
Yes (n = 72)	No (n = 253)				*p*
4.47 ± 0.02	4.41 ± 0.02				0.13
**Provision of finished feed (concentrate) to adult animals**					
Yes (n = 321)	No (n = 4)				*p*
4.43 ± 0.01	4.45 ± 0.16				0.83
**Provision of finished feed (concentrate) to adult animals throughout the year**					
Yes (n = 219)	No (n = 106)				*p*
4.45 ± 0.02	4.37 ± 0.03				0.005
**Type of finished feed (concentrate) to adult animals**					
Mash (n = 122)	Pelleted (n = 101)	Flakes (n = 4)	Crumbled (n = 87)	Other (n = 7)	*p*
4.45 ± 0.02	4.40 ± 0.03	4.36 ± 0.10	4.43 ± 0.02	4.35 ± 0.09	0.61
**Average quantity of finished feed (concentrate) provided daily to animals during the preceding season**					
≤ 0.6 kg (n = 140)	0.61 – 1.2 kg (n = 148)	> 1.2 kg (n = 37)			*p*
4.40 ± 0.02	4.43 ± 0.02	4.48 ± 0.05			0.24
**Age of farmer**					
Up to 50 years (n = 197)	Over 50 years (n = 128)				*p*
4.44 ± 0.02	4.41 ± 0.02				0.39
**Length of previous animal farming experience**					
≤ 5 years (n = 74)	> 5 years (n = 251)				*p*
4.45 ± 0.03	4.42 ± 0.02				0.28
**General education of farmer**					
Primary (n = 57)	Secondary and post-secondary (n = 225)	Tertiary (n = 43)			*p*
4.52 ± 0.03	4.40 ± 0.02	4.44 ± 0.05			0.004

**Farmer by profession**		
Yes (n = 292)	No (n = 33)	*p*
4.43 ± 0.01	4.38 ± 0.08	0.29
**Family tradition in farming**		
Yes (n = 283)	No (n = 42)	*p*
4.42 ± 0.02	4.49 ± 0.04	0.09
**Presence of working staff in the farm**		
Yes (n = 123)	No (n = 202)	*p*
4.45 ± 0.02	4.41 ± 0.02	0.21

Table S6. Effects of husbandry- and human resources-related factors (n = 43) in the fat content (%) in the bulk-tank raw milk of 119 goat herds in Greece.

**Management system applied in the farm**				
Intensive (n = 9)	Semi-intensive (n = 29)	Semi-extensive (n = 61)	Extensive (n = 20)	*p*
4.53 ± 0.43	4.66 ± 0.21	4.69 ± 0.13	5.27 ± 0.38	0.24
**Month into the lactation period at sampling**				
0 – 1st (n = 8)	2nd – 5th (n = 60)	6th – 9th (n = 43)	After 9th (n = 8)	*p*
5.41 ± 0.55	4.93 ± 0.16	4.58 ± 0.15	3.94 ± 0.47	0.05
**Availability of straw bedding**				
Yes (n = 76)	No (n = 43)			*p*
4.66 ± 0.14	4.96 ± 0.19			0.20
**Availability of mechanical ventilators**				
Yes (n = 8)	No (n = 111)			*p*
4.76 ± 0.51	4.77 ± 0.11			0.98
**Grazing practiced**				
Yes (n = 113)	No (n = 6)			*p*
4.79 ± 0.11	4.47 ± 0.60			0.54
**Grazing land available to animals**				
≤ 0.50 ac. per animal (n = 17)	0.51-2.00 ac. per animal (n = 31)	> 2.00 ac. per animal (n = 71)		*p*
4.51 ± 0.29	5.11 ± 0.23	4.68 ± 0.14		0.17
**Availability of milking parlour**				
Yes (n = 66)	No (n = 53)			*p*
4.52 ± 0.13	4.08 ± 0.19			0.012
**Number of milking units in the parlour**				
< 24 (n = 37)	24 (n = 26)	> 24 (n = 3)		*p*
4.51 ± 0.17	4.57 ± 0.22	4.26 ± 0.44		0.88
**Number of available milking units per animal position**				
< 1 (n = 42)	1 (n = 24)			*p*
4.54 ± 0.17	4.49 ± 0.19			0.85
**System pressure**				
< 38 kPa (n = 5)	38 - 42 kPa (n = 55)	> 42 kPa (n = 6)		*p*
4.76 ± 0.45	4.40 ± 0.14	5.49 ± 0.27		0.040
**Type of flow line**				
High (n = 43)	Low (n = 16)	Other (n = 7)		*p*
4.38 ± 0.13	4.59 ± 0.30	5.27 ± 0.57		0.11
**No. of does in the herd**				
≤ 165 does (n = 56)	166-330 does (n = 37)	331-500 does (n = 13)	> 500 does (n = 13)	*p*
4.63 ± 0.15	5.06 ± 0.23	4.43 ± 0.30	4.85 ± 0.31	0.27
**Breed of does**				
Alpine (n = 9)	Crossbreeds (n = 18)	Damascus (n = 18)	Kefallinia (n = 1)	*p*
3.90 ± 0.30	4.54 ± 0.35	4.76 ± 0.17	5.72	0.15
Local (*Capra prisca*) (n = 50)	Murcia (n = 13)	Saanen (n = 5)	Skopelos (n = 5)	
4.90 ± 0.18	5.08 ± 0.30	4.40 ± 0.59	5.24 ± 0.72	

**Average age of culling does**			
≤ 6 years (n = 45)	> 6 years (n = 74)		*p*
4.68 ± 0.17	4.83 ± 0.15		0.52
**Total milk quantity per doe obtained during the preceding milking period**			
≤ 200 L (n = 73)	201 - 400 L (n = 32)	> 400 L (n = 14)	*p*
4.89 ± 0.15	4.58 ± 0.17	4.53 ± 0.34	0.37
**Average number of kids born per doe**			
≤ 1.50 (n = 102)	> 1.50 (n = 17)		*p*
4.82 ± 0.12	4.44 ± 0.23		0.22
**Collaboration with a veterinarian**			
Yes (n = 101)	No (n = 18)		*p*
4.84 ± 0.12	4.37 ± 0.24		0.13
**Clinical mastitis annual incidence risk in the herd**			
≤ 0.50% (n = 48)	> 0.50% (n = 71)		*p*
4.71 ± 0.19	4.81 ± 0.14		0.69
**Application of reproductive control practices in the farm**			
Yes (n = 17)	No (n = 102)		*p*
4.83 ± 0.26	4.76 ± 0.12		0.83
**Nutritional modifications performed according to the reproductive stage**			
Yes (n = 68)	No (n = 51)		*p*
4.62 ± 0.14	4.97 ± 0.18		0.11
**Method for drying-off at the end of the lactation period**			
Abrupt (n = 5)	Progressive (n = 114)		*p*
4.13 ± 0.55	4.80 ± 0.11		0.23
**Age of kid removal from their dams**			
< 45 days (n = 26)	45 – 60 days (n = 44)	> 60 days (n = 49)	*p*
4.43 ± 0.24	4.59 ± 0.17	5.11 ± 0.18	0.032
**Daily number of milking sessions**			
One (n = 4)	Two (n = 108)	Three (n = 7)	*p*
4.12 ± 0.69	4.78 ± 0.12	5.01 ± 0.33	0.50
**Number of feet care sessions provided to the does annually**			
No feet care provided (n = 48)	1 - 2 (n = 69)	> 2 (n = 2)	*p*
4.91 ± 0.19	4.68 ± 0.14	4.47 ± 0.91	0.58
**Shearing of animals**			
Yes (n = 102)	No (n = 17)		*p*
4.75 ± 0.12	4.86 ± 0.29		0.75
**Vaccination against mastitis**			
Yes (n = 35)	No (n = 84)		*p*
4.69 ± 0.17	4.80 ± 0.14		0.65
**Vaccination against contagious agalactia**			
Yes (n = 65)	No (n = 54)		*p*
4.78 ± 0.16	4.75 ± 0.16		0.89

Administration of anthelmintic treatment during the last stage of pregnancy

Yes (n = 75)	No (n = 44)	*p*
4.88 ± 0.15	4.58 ± 0.16	0.19

Duration of grazing during the year

No grazing (n = 8)	1 - 5 months (n = 13)	6 - 10 months (n = 33)	11 - 12 months (n = 65)	*p*
4.51 ± 0.44	4.58 ± 0.39	4.58 ± 0.20	4.94 ± 0.15	0.44

Provision of hay as fodder to animals

Yes (n = 116)	No (n = 3)	*p*
4.77 ± 0.11	4.87 ± 0.37	0.89

Average quantity of hay provided daily to animals during the preceding season

≤ 0.6 kg (n = 59)	> 0.6 kg (n = 60)	*p*
4.97 ± 0.17	4.58 ± 0.14	0.08

Provision of straw to animals

Yes (n = 73)	No (n = 46)	*p*
4.65 ± 0.13	4.95 ± 0.21	0.20

Provision of silage to adult animals

Yes (n = 18)	No (n = 101)	*p*
4.59 ± 0.23	4.80 ± 0.12	0.50

Provision of finished feed (concentrate) to adult animals

Yes (n = 116)	No (n = 3)	*p*
4.76 ± 0.11	5.30 ± 0.09	0.45

Provision of finished feed (concentrate) to adult animals throughout the year

Yes (n = 70)	No (n = 49)	*p*
4.54 ± 0.13	5.05 ± 0.20	0.020

Type of finished feed (concentrate) to adult animals

Mash (n = 43)	Pelleted (n = 34)	Flakes (n = 0)	Crumbled (n = 34)	Other (n = 5)	*p*
4.78 ± 0.19	4.71 ± 0.19	-	4.89 ± 0.23	4.48 ± 0.46	0.84

Average quantity of finished feed (concentrate) provided daily to animals during the preceding season

≤ 0.6 kg (n = 48)	0.61 – 1.2 kg (n = 35)	> 1.2 kg (n = 36)	*p*
4.87 ± 0.20	4.73 ± 0.19	4.68 ± 0.19	0.75

Age of farmer

Up to 50 years (n = 73)	Over 50 years (n = 46)	*p*
4.73 ± 0.15	4.83 ± 0.17	0.67

Length of previous animal farming experience

≤ 5 years (n = 24)	> 5 years (n = 95)	*p*
4.75 ± 0.26	4.77 ± 0.12	0.94

General education of farmer

Primary (n = 20)	Secondary and post-secondary (n = 89)	Tertiary (n = 10)	*p*
5.26 ± 0.32	4.72 ± 0.12	4.24 ± 0.34	0.07

Farmer by profession

Yes (n = 105)	No (n = 14)	*p*
4.82 ± 0.12	4.42 ± 0.25	0.25

**Family tradition in farming**		
Yes (n = 104)	No (n = 15)	*p*
4.73 ± 0.12	5.02 ± 0.32	0.40
**Presence of working staff in the farm**		
Yes (n = 34)	No (n = 85)	*p*
4.60 ± 0.20	4.84 ± 0.14	0.35

Table S7. Effects of husbandry- and human resources-related factors (n = 43) in the protein content (%) in the bulk-tank raw milk of 119 goat herds in Greece.

**Management system applied in the farm**				
Intensive (n = 9)	Semi-intensive (n = 29)	Semi-extensive (n = 61)	Extensive (n = 20)	*p*
3.29 ± 0.10	3.19 ± 0.05	3.25 ± 0.05	3.18 ± 0.05	0.70
**Month into the lactation period at sampling**				
0 – 1st (n = 8)	2nd – 5th (n = 60)	6th – 9th (n = 43)	After 9th (n = 8)	*p*
3.39 ± 0.05	3.28 ± 0.04	3.13 ± 0.04	3.17 ± 0.07	0.030
**Availability of straw bedding**				
Yes (n = 76)		No (n = 43)		*p*
3.23 ± 0.04		3.23 ± 0.05		0.99
**Availability of mechanical ventilators**				
Yes (n = 8)		No (n = 111)		*p*
3.33 ± 0.11		3.22 ± 0.03		0.33
**Grazing practiced**				
Yes (n = 113)		No (n = 6)		*p*
3.23 ± 0.03		3.22 ± 0.08		0.92
**Grazing land available to animals**				
≤ 0.50 ac. per animal (n = 17)	0.51-2.00 ac. per animal (n = 31)	> 2.00 ac. per animal (n = 71)		*p*
3.20 ± 0.05	3.22 ± 0.04	3.24 ± 0.04		0.88
**Availability of milking parlour**				
Yes (n = 66)		No (n = 53)		*p*
3.22 ± 0.04		3.24 ± 0.04		0.78
**Number of milking units in the parlour**				
< 24 (n = 37)	24 (n = 26)	> 24 (n = 3)		*p*
3.21 ± 0.05	3.25 ± 0.08	3.16 ± 0.20		0.85
**Number of available milking units per animal position**				
< 1 (n = 42)		1 (n = 24)		*p*
3.23 ± 0.05		3.20 ± 0.08		0.70
**System pressure**				
< 38 kPa (n = 5)	38 - 42 kPa (n = 55)	> 42 kPa (n = 6)		*p*
3.27 ± 0.15	3.20 ± 0.05	3.34 ± 0.14		0.61
**Type of flow line**				
High (n = 43)	Low (n = 16)	Other (n = 7)		*p*
3.18 ± 0.05	3.20 ± 0.06	3.49 ± 0.12		0.07
**No. of does in the herd**				
≤ 165 does (n = 56)	166-330 does (n = 37)	331-500 does (n = 13)	> 500 does (n = 13)	*p*
3.28 ± 0.05	3.19 ± 0.03	3.14 ± 0.05	3.19 ± 0.06	0.33
**Breed of does**				
Alpine (n = 9)	Crossbreeds (n = 18)	Damascus (n = 18)	Kefallinia (n = 1)	*p*
3.08 ± 0.10	3.38 ± 0.08	3.14 ± 0.04	3.08	0.15
Local (*Capra prisca*) (n = 50)	Murcia (n = 13)	Saanen (n = 5)	Skopelos (n = 5)	
3.17 ± 0.03	3.37 ± 0.13	3.42 ± 0.25	3.32 ± 0.10	

**Average age of culling does**			
≤ 6 years (n = 45)	> 6 years (n = 74)		*p*
3.21 ± 0.04	3.24 ± 0.04		0.70
**Total milk quantity per doe obtained during the preceding milking period**			
≤ 200 L (n = 73)	201 - 400 L (n = 32)	> 400 L (n = 14)	*p*
3.22 ± 0.03	3.23 ± 0.06	3.31 ± 0.13	0.70
**Average number of kids born per doe**			
≤ 1.50 (n = 102)	> 1.50 (n = 17)		*p*
3.23 ± 0.03	3.23 ± 0.10		0.98
**Collaboration with a veterinarian**			
Yes (n = 101)	No (n = 18)		*p*
3.22 ± 0.03	3.30 ± 0.11		0.29
**Clinical mastitis annual incidence risk in the herd**			
≤ 0.50% (n = 48)	> 0.50% (n = 71)		*p*
3.21 ± 0.05	3.24 ± 0.04		0.66
**Application of reproductive control practices in the farm**			
Yes (n = 17)	No (n = 102)		*p*
3.24 ± 0.10	3.23 ± 0.03		0.91
**Nutritional modifications performed according to the reproductive stage**			
Yes (n = 68)	No (n = 51)		*p*
3.22 ± 0.03	3.24 ± 0.05		0.65
**Method for drying-off at the end of the lactation period**			
Abrupt (n = 5)	Progressive (n = 114)		*p*
3.16 ± 0.08	3.23 ± 0.03		0.60
**Age of kid removal from their dams**			
< 45 days (n = 26)	45 – 60 days (n = 44)	> 60 days (n = 49)	*p*
3.24 ± 0.06	3.23 ± 0.05	3.22 ± 0.04	0.97
**Daily number of milking sessions**			
One (n = 4)	Two (n = 108)	Three (n = 7)	*p*
3.27 ± 0.12	3.23 ± 0.03	3.14 ± 0.08	0.72
**Number of feet care sessions provided to the does annually**			
No feet care provided (n = 48)	1 - 2 (n = 69)	> 2 (n = 2)	*p*
3.22 ± 0.04	3.23 ± 0.04	3.24 ± 0.19	0.99
**Shearing of animals**			
Yes (n = 102)	No (n = 17)		*p*
3.24 ± 0.03	3.18 ± 0.05		0.47
**Vaccination against mastitis**			
Yes (n = 35)	No (n = 84)		*p*
3.18 ± 0.06	3.25 ± 0.03		0.30
**Vaccination against contagious agalactia**			
Yes (n = 65)	No (n = 54)		*p*
3.24 ± 0.04	3.21 ± 0.04		0.63

**Administration of anthelmintic treatment during the last stage of pregnancy**					
Yes (n = 75)	No (n = 44)				*p*
3.23 ± 0.03	3.22 ± 0.05				0.91
**Duration of grazing during the year**					
No grazing (n = 8)	1 - 5 months (n = 13)	6 - 10 months (n = 33)	11 - 12 months (n = 65)		*p*
3.27 ± 0.07	3.48 ± 0.12	3.21 ± 0.06	3.18 ± 0.03		0.014
**Provision of hay as fodder to animals**					
Yes (n = 116)	No (n = 3)				*p*
3.23 ± 0.03	2.99 ± 0.02				0.18
**Average quantity of hay provided daily to animals during the preceding season**					
≤ 0.6 kg (n = 59)	> 0.6 kg (n = 60)				*p*
3.19 ± 0.03	3.26 ± 0.05				0.20
**Provision of straw to animals**					
Yes (n = 73)	No (n = 46)				*p*
3.22 ± 0.04	3.24 ± 0.05				0.77
**Provision of silage to adult animals**					
Yes (n = 18)	No (n = 101)				*p*
3.34 ± 0.07	3.21 ± 0.03				0.09
**Provision of finished feed (concentrate) to adult animals**					
Yes (n = 116)	No (n = 3)				*p*
3.23 ± 0.03	3.14 ± 0.21				0.61
**Provision of finished feed (concentrate) to adult animals throughout the year**					
Yes (n = 70)	No (n = 49)				*p*
3.21 ± 0.03	3.25 ± 0.05				0.43
**Type of finished feed (concentrate) to adult animals**					
Mash (n = 43)	Pelleted (n = 34)	Flakes (n = 0)	Crumbled (n = 34)	Other (n = 5)	*p*
3.26 ± 0.04	3.21 ± 0.05	-	3.23 ± 0.06	3.14 ± 0.08	0.73
**Average quantity of finished feed (concentrate) provided daily to animals during the preceding season**					
≤ 0.6 kg (n = 48)	0.61 – 1.2 kg (n = 35)	> 1.2 kg (n = 36)			*p*
3.20 ± 0.04	3.25 ± 0.06	3.25 ± 0.05			0.67
**Age of farmer**					
Up to 50 years (n = 73)	Over 50 years (n = 46)				*p*
3.23 ± 0.04	3.22 ± 0.04				0.91
**Length of previous animal farming experience**					
≤ 5 years (n = 24)	> 5 years (n = 95)				*p*
3.22 ± 0.05	3.23 ± 0.03				0.82
**General education of farmer**					
Primary (n = 20)	Secondary and post-secondary (n = 89)	Tertiary (n = 10)			*p*
3.26 ± 0.05	3.22 ± 0.03	3.24 ± 0.10			0.84
**Farmer by profession**					
Yes (n = 105)	No (n = 14)				*p*
3.23 ± 0.03	3.23 ± 0.09				0.99

**Family tradition in farming**		
Yes (n = 104)	No (n = 15)	*p*
3.23 ± 0.03	3.23 ± 0.06	0.94
**Presence of working staff in the farm**		
Yes (n = 34)	No (n = 85)	*p*
3.27 ± 0.06	3.21 ± 0.03	0.37

Table S8. Results of univariable analysis of variables (*n* = 32) for evaluation of the outcome ''fat and protein content in bulk-tank milk concurrently above the average contents found for all flocks'' in 325 sheep flocks in Greece.

Flocks with fat or protein content in bulk-tank milk concurrently below the average content found for all flocks				Flocks with fat and protein content in bulk-tank milk concurrently above the average contents found for all flocks				
**Average somatic cell counts in bulk-tank raw milk**								
								p
0.511×10^6 cells mL^{-1}				0.411×10^6 cells mL^{-1}				0.020
**Average total bacterial counts in bulk-tank raw milk**								
								p
418×10^3 cfu mL^{-1}				341×10^3 cells mL^{-1}				0.34
**Staphylococcal recovery from bulk-tank raw milk**								
No staphylococcal isolation		Staphylococcal isolation		No staphylococcal isolation		Staphylococcal isolation		*p*
88		158		32		47		0.45
**epg counts in pooled faecal samples**								
≤ 300 epg		≥ 350 epg		≤ 300 epg		≥ 350 epg		*p*
190		56		69		10		0.05
**Proportion of *Teladorsagia* in pooled faecal samples**								
0%	1%-63%	≥ 64%		0%	1%-63%	≥ 64%		*P*
37	137	72		10	43	26		0.77
**Management system applied in farms**								
Intensive (n = 43)	Semi-intensive (n = 151)	Semi-extensive (n = 107)	Extensive (n = 24)	Intensive (n = 43)	Semi-intensive (n = 151)	Semi-extensive (n = 107)	Extensive (n = 24)	*p*
33	106	87	20	10	45	20	4	0.16
**Month into the lactation period at sampling**								
0 – 1st (n = 23)	2nd – 5th (n = 138)	6th – 9th (n = 147)	After 9th (n = 17)	0 – 1st (n = 23)	2nd – 5th (n = 138)	6th – 9th (n = 147)	After 9th (n = 17)	*p*
21	93	117	15	2	45	30	2	0.012
**Availability of straw bedding**								
Yes (n = 268)		No (n = 57)		Yes (n = 268)		No (n = 57)		*p*
201		45		67		12		0.53
**Grazing practiced**								
Yes (n = 281)		No (n = 44)		Yes (n = 281)		No (n = 44)		*p*
211		35		70		9		0.52
**Grazing land available to animals**								

≤ 0.50 ac. per animal (n = 118)	0.51-2.00 ac. per animal (n = 130)	> 2.00 ac. per animal (n = 77)	≤ 0.50 ac. per animal (n = 118)	0.51-2.00 ac. per animal (n = 130)	> 2.00 ac. per animal (n = 77)			*P*
91	97	58	27	33	19			0.90
**Availability of milking parlour**								
Yes (n = 255)	No (n = 70)	Yes (n = 255)	No (n = 70)					*p*
195	51	60	19					0.53
**Number of available milking units per animal position**								
< 1 (n = 177)	1 (n = 78)	< 1 (n = 177)	1 (n = 78)					*p*
134	61	43	17					0.66
**System pressure**								
< 38 kPa (n = 22)	38 - 42 kPa (n = 203)	> 42 kPa (n = 30)	< 38 kPa (n = 22)	38 - 42 kPa (n = 203)	> 42 kPa (n = 30)			*p*
18	154	23	4	49	7			0.82
**Type of flow line**								
High (n = 182)	Low (n = 55)	Other (n = 18)	High (n = 182)	Low (n = 55)	Other (n = 18)			*p*
135	47	13	47	8	5			0.20
**No. of ewes in the flock**								
≤ 165 ewes (n = 88)	166 - 330 ewes (n = 120)	331 - 500 ewes (n = 66)	> 500 ewes (n = 51)	≤ 165 ewes (n = 88)	166 - 330 ewes (n = 120)	331 - 500 ewes (n = 66)	> 500 ewes (n = 51)	*p*
65	89	52	40	23	31	14	11	0.83
**Breed of ewes**								
Assaf (n = 30)	Awassi (n = 1)	Boutsko (n = 2)	Chios (n = 44)	Assaf (n = 30)	Awassi (n = 1)	Boutsko (n = 2)	Chios (n = 44)	*p*
25	1	1	33	5	0	1	11	0.47
Crossbreeds (n = 43)	Friesarta (n = 12)	Friesian (n = 13)	Karagouniko (n = 5)	Crossbreeds (n = 43)	Friesarta (n = 12)	Friesian (n = 13)	Karagouniko (n = 5)	
32	11	10	4	11	1	3	1	
Kefallinia (n = 1)	Lacaune (n = 95)	Local (n = 55)	Mytilini (n = 18)	Kefallinia (n = 1)	Lacaune (n = 95)	Local (n = 55)	Mytilini (n = 18)	
0	64	44	15	1	31	11	3	
Sfakia (n = 6)				Sfakia (n = 6)				
5				1				
**Average number of lambs born per ewe**								
≤ 1.50 (n = 280)	> 1.50 (n = 45)	≤ 1.50 (n = 80)	> 1.50 (n = 45)					*p*
215	31	65	14					0.25
**Collaboration with a veterinarian**								
Yes (n = 277)	No (n = 48)	Yes (n = 277)	No (n = 48)					*p*
204	42	73	6					0.039
**Nutritional modifications performed according to the reproductive stage**								

Yes (n = 229)	No (n = 96)	Yes (n = 229)	No (n = 96)	*p*
170	76	59	20	0.34

Age of lamb removal from their dams

< 45 days (n = 119)	45 – 60 days (n = 170)	> 60 days (n = 36)	< 45 days (n = 119)	45 – 60 days (n = 170)	> 60 days (n = 36)	*p*
92	125	29	27	45	7	0.59

Daily number of milking sessions

One (n = 1)	Two (n = 264)	Three (n = 60)	One (n = 1)	Two (n = 264)	Three (n = 60)	*p*
0	199	47	1	65	13	0.19

Number of feet care sessions provided to the ewes annually

No feet care provided (n = 102)	1 - 2 (n = 203)	> 2 (n = 20)	No feet care provided (n = 102)	1 - 2 (n = 203)	> 2 (n = 20)	*p*
77	153	16	25	50	4	0.90

Administration of anthelmintic treatment during the last stage of pregnancy

Yes (n = 224)	No (n = 101)	Yes (n = 223)	No (n = 102)	*p*
165	81	59	20	0.20

Duration of grazing during the year

No grazing (n = 44)	1 - 5 months (n = 46)	6 - 10 months (n = 124)	11 - 12 months (n = 111)	No grazing (n = 44)	1 - 5 months (n = 46)	6 - 10 months (n = 124)	11 - 12 months (n = 111)	*p*
35	34	86	91	9	12	38	20	0.14

Average quantity of hay provided daily to animals during the preceding season

≤ 0.6 kg (n = 109)	> 0.6 kg (n = 216)	≤ 0.6 kg (n = 109)	> 0.6 kg (n = 216)	*p*
85	161	24	55	0.49

Provision of silage to adult animals

Yes (n = 72)	No (n = 253)	Yes (n = 72)	No (n = 253)	*p*
52	194	20	59	0.44

Provision of finished feed (concentrate) to adult animals throughout the year

Yes (n = 219)	No (n = 106)	Yes (n = 219)	No (n = 106)	*p*
164	82	55	24	0.63

Type of finished feed (concentrate) to adult animals

Mash (n = 122)	Pelleted (n = 101)	Flakes (n = 4)	Crumbled (n = 87)	Other (n = 7)	Mash (n = 122)	Pelleted (n = 101)	Flakes (n = 4)	Crumbled (n = 87)	Other (n = 7)	*p*
85	82	3	67	6	37	19	1	20	1	0.34

**Length of previous animal farming experience**						
≤ 5 years (n = 74)	> 5 years (n = 251)	≤ 5 years (n = 74)	> 5 years (n = 251)			*p*
57	189	17	62			0.76
**General education of farmer**						
Primary (n = 57)	Secondary and post-secondary (n = 225)	Tertiary (n = 43)	Primary (n = 57)	Secondary and post-secondary (n = 225)	Tertiary (n = 43)	*p*
36	179	31	21	46	12	0.030
**Family tradition in farming**						
Yes (n = 283)	No (n = 42)	Yes (n = 283)	No (n = 42)			*p*
213	33	70	9			0.64
**Presence of working staff in the farm**						
Yes (n = 123)	No (n = 202)	Yes (n = 123)	No (n = 202)			*p*
94	152	29	50			0.81

Table S9. Results of univariable analysis of variables (n = 19) for evaluation of the outcome ''fat and protein content in bulk-tank milk concurrently above the average contents found for all flocks'' in 119 goat herds in Greece.

Herds with fat or protein content in bulk-tank milk concurrently below the average content found for all herds				Herds with fat and protein content in bulk-tank milk concurrently above the average contents found for all herds				
**Average somatic cell counts in bulk-tank raw milk**								
								p
0.904 × 10^6 cells mL^{-1}				0.683 × 10^6 cells mL^{-1}				0.015
**Average total bacterial counts in bulk-tank raw milk**								
								p
624 × 10^3 cfu mL^{-1}				528 × 10^3 cfu mL^{-1}				0.59
**Proportion of *Teladorsagia* in pooled faecal samples**								
0%	1%-64%	≥ 65%		0%	1%-64%	≥ 65%		*P*
9	42	36		4	24	4		0.012
**Month into the lactation period at sampling**								
0 – 1st (n = 8)	2nd – 5th (n = 60)	6th – 9th (n = 43)	After 9th (n = 8)	0 – 1st (n = 8)	2nd – 5th (n = 60)	6th – 9th (n = 43)	After 9th (n = 8)	*p*
2	42	36	7	6	18	7	1	0.005
**Grazing land available to animals**								
≤ 0.50 ac. per animal (n = 17)	0.51-2.00 ac. per animal (n = 31)	> 2.00 ac. per animal (n = 71)		≤ 0.50 ac. per animal (n = 17)	0.51-2.00 ac. per animal (n = 31)	> 2.00 ac. per animal (n = 71)		*p*
12	23	52		5	8	19		0.96
**Availability of milking parlour**								
Yes (n = 66)	No (n = 53)			Yes (n = 66)	No (n = 53)			*p*
50	37			16	16			0.47
**System pressure**								
< 38 kPa (n = 5)	38 - 42 kPa (n = 55)	> 42 kPa (n = 6)		< 38 kPa (n = 5)	38 - 42 kPa (n = 55)	> 42 kPa (n = 6)		*p*
4	43	3		1	12	3		0.30
**Type of flow line**								
High (n = 43)	Low (n = 16)	Other (n = 7)		High (n = 43)	Low (n = 16)	Other (n = 7)		*p*
37	11	2		6	5	5		0.003
**Breed of does**								
Alpine (n = 9)	Crossbreeds (n = 18)	Damascus (n = 18)	Kefallinia (n = 1)	Alpine (n = 9)	Crossbreeds (n = 18)	Damascus (n = 18)	Kefallinia (n = 1)	*p*
8	11	17	1	1	7	1	0	0.029
Local (*Capra prisca*) (n = 50)	Murcia (n = 13)	Saanen (n = 5)	Skopelos (n = 5)	Local (*Capra prisca*) (n = 50)	Murcia (n = 13)	Saanen (n = 5)	Skopelos (n = 5)	
39	6	3	2	11	7	2	3	

**Collaboration with a veterinarian**								
Yes (n = 101)	No (n = 18)	Yes (n = 101)	No (n = 18)					*p*
4	14	28	4					0.63
**Nutritional modifications performed according to the reproductive stage**								
Yes (n = 68)	No (n = 51)	Yes (n = 68)	No (n = 51)					*p*
54	33	14	18					0.07
**Age of kid removal from their dams**								
< 45 days (n = 26)	45 – 60 days (n = 44)	> 60 days (n = 49)	< 45 days (n = 26)	45 – 60 days (n = 44)	> 60 days (n = 49)			*p*
20	32	35	6	12	14			0.88
**Administration of anthelmintic treatment during the last stage of pregnancy**								
Yes (n = 75)	No (n = 44)	Yes (n = 75)	No (n = 44)					*p*
55	32	24	8					0.23
**Duration of grazing during the year**								
No grazing (n = 8)	1 - 5 months (n = 13)	6 - 10 months (n = 33)	11 - 12 months (n = 65)	No grazing (n = 8)	1 - 5 months (n = 13)	6 - 10 months (n = 33)	11 - 12 months (n = 65)	*p*
5	8	25	49	3	5	8	16	0.65
**Provision of hay as fodder to animals**								
Yes (n = 116)	No (n = 3)	Yes (n = 116)	No (n = 3)					*p*
84	3	32	0					0.29
**Average quantity of hay provided daily to animals during the preceding season**								
≤ 0.6 kg (n = 59)	> 0.6 kg (n = 60)	≤ 0.6 kg (n = 59)	> 0.6 kg (n = 60)					*p*
44	43	15	17					0.72
**Provision of silage to adult animals**								
Yes (n = 18)	No (n = 101)	Yes (n = 18)	No (n = 101)					*p*
12	75	6	26					0.50
**Provision of finished feed (concentrate) to adult animals throughout the year**								
Yes (n = 70)	No (n = 49)	Yes (n = 70)	No (n = 49)					*p*
52	35	18	14					0.73
**General education of farmer**								
Primary (n = 20)	Secondary and post-secondary (n = 89)	Tertiary (n = 10)	Primary (n = 20)	Secondary and post-secondary (n = 89)	Tertiary (n = 10)			*p*
14	64	9	6	25	1			0.45
